# Defective inducible T cell costimulator signalling drives neuropilin-1^hi^ CD4^+^ T cell-mediated non-germinal centre B cell activation via impaired regulatory T cell maintenance in a lupus model

**DOI:** 10.1038/s12276-026-01756-6

**Published:** 2026-06-10

**Authors:** Sumin Kang, Da Eun Kim, Nahyun Kim, Eunju O, Hye Jin Yoon, Yesung Joo, Sung-Wook Hong, Kwang Soon Kim

**Affiliations:** 1 Department of Life Sciences, Pohang University of Science and Technology (POSTECH), Pohang, Republic of Korea; 2 Department of Integrative Biosciences and Biotechnology, Pohang University of Science and Technology (POSTECH), Pohang, Republic of Korea; 3 Department of Biotechnology, College of Life Science and Biotechnology, Yonsei University, Seoul, Republic of Korea

**Keywords:** Autoimmunity, Lymphocyte differentiation, Systemic lupus erythematosus

## Abstract

Inducible T cell costimulator (ICOS) signalling is essential for follicular helper T (Tfh) cell activation and has emerged as a therapeutic target in B cell-dependent autoimmune diseases such as systemic lupus erythematosus (SLE). However, its dual role in supporting both Foxp3^+^ regulatory T (Treg) cells and Tfh cell responses complicates therapeutic interventions. Here, we demonstrate that disruption of ICOS signalling in *Sanroque* mice impairs Treg cell maintenance, unleashing pathogenic T cell responses. In ICOS ligand-deficient *Sanroque* mice, defective ICOS signalling disrupts canonical Tfh cell-dependent germinal centre (GC) responses but fails to prevent autoantibody production and reverse SLE-like symptoms. Notably, Treg cell reduction in ICOS ligand-deficient *Sanroque* mice is associated with augmentation of self-reactive neuropilin-1 (Nrp-1)^hi^ CD4^+^ conventional T (Tconv) cells. Mechanistically, Nrp-1^hi^ CD4^+^ Tconv cells effectively produce IFN-γ and outcompete Treg cells for dendritic cell engagement. Nrp-1^hi^ CD4^+^ Tconv cells acquire PD-1^hi^ CXCR5^−^ peripheral helper T (Tph) cell phenotype, and drive non-GC B cell responses by promoting the induction of age-associated B cells through contact-dependent mechanisms in the absence of Treg cells. Our findings highlight that ICOS signalling orchestrates GC and non-GC B cell responses in SLE via impaired Treg cell maintenance, and also underscore potential risks in therapeutic strategies targeting ICOS signalling for SLE, as it could inadvertently promote T cell-mediated inflammation and non-GC B cell responses through the induction of age-associated B cells.

## Defective Inducible T Cell Costimulator Signalling Promotes Non-Germinal Centre Autoimmunity

Systemic lupus erythematosus (SLE) is a complex autoimmune disorder driven by dysregulated B and T cell responses. This study explores the role of regulatory T (Treg) cells and their interaction with other immune cells in SLE, using *Sanroque* mice as a model. Researchers found that Treg cells expand, maintaining suppressive function, yet fail to fully control autoimmunity. Defective inducible T cell costimulator signalling reduces Treg cells and disrupts germinal centre (GC) B cell responses but does not prevent autoantibody production, suggesting that non-GC B cell responses are involved. Notably, Nrp-1^hi^ CD4^+^ T cells, which are self-reactive, outcompete Treg cells for dendritic cell engagement, and promote age-associated B cells, contributing to autoantibody production. These findings suggest that targeting inducible T cell costimulator signalling in SLE may have dual effects, potentially reducing Treg cells and activating non-GC responses. Future research could explore therapeutic strategies targeting Nrp-1^hi^ CD4^+^ T cells to manage SLE more effectively.

This summary was initially drafted using artificial intelligence, then revised and fact-checked by the author.

## Introduction

Systemic lupus erythematosus (SLE) is a chronic autoimmune disorder characterized by dysregulated immune responses against self-antigens, resulting in systemic inflammation and damage across multiple organs such as the lung, liver and kidney. A hallmark of SLE is the dysregulation of B cells, which results in the production of autoreactive IgG antibodies such as IgG specific to nuclear self-antigens^1^. The aberrant production of autoreactive IgG antibodies in SLE, as observed in both human patients and murine lupus models, is closely associated with the augmentation of PD-1^hi^ CXCR5^+^ follicular helper T (Tfh) cells and spontaneous germinal centre (GC) B cell responses^2^. However, emerging evidence suggests that non-GC B cell responses, an alternative pathway of B cell activation, which are promoted by CD4^+^ T cells other than Tfh cells, also significantly contribute to SLE pathogenesis^3^. This highlights the heterogeneity of SLE, which may manifest with GC- or non-GC-dominant features, underscoring distinct immune activation pathways in SLE.

*Sanroque* mice serve as a valuable model for exploring SLE mechanisms^4,5^. These mice carry a single amino acid substitution in Roquin-1, a RING-type ubiquitin ligase encoded by the *Rc3h1* gene. This mutation abnormally increases the stability of mRNA encoding IFN-γ and inducible T cell costimulator (ICOS)^6,7^, which are essential for Th1 and Tfh cells, respectively. Roquin-1 and its paralog Roquin-2 redundantly promote the decay of target mRNAs. In *Sanroque* mice, the mutated Roquin-1 acts as a dominant negative mutant, thereby increasing the stability of target mRNAs^5–8^. Consequently, *Sanroque* mice exhibit excessive Th1 and Tfh cell populations, increased tissue infiltration by activated CD4^+^ T cells and heightened autoreactive IgG production, which collectively contribute to organ damage^4,9,10^. Although enhanced ICOS signalling and the augmentation of Tfh cells are closely associated with SLE pathogenesis in *Sanroque* mice^11^, it has been shown that deficiency of ICOS signalling and the absence of Tfh cells do not fully reverse the SLE-like symptoms^6,12^.

ICOS, a member of the CD28 superfamily, plays a dual role in T cell regulation: it supports the induction of Tfh cells^13,14^ and the maintenance of Foxp3^+^ regulatory CD4^+^ T (Treg) cells^15,16^, which are essential for immune tolerance and prevention of autoimmune disease^17,18^. Intriguingly, *Sanroque* mice exhibit increased Treg cell levels during the onset of SLE-like disease^6,19^. In SLE patients, however, the role of Treg cells is variable, with some patients displaying reduced Treg cell numbers and impaired suppressive functions, and others retaining functional Treg cells^20,21^. Considering the role of ICOS signalling in Treg cell maintenance, it has been proposed that the failure to reverse SLE-like symptoms in *Sanroque* mice with ICOS deficiency is due to the reduction of Treg cells^6^. This variability raises the question of how Treg cells contribute to SLE heterogeneity, particularly in relation to GC- and non-GC-dominant phenotypes. Specifically, it remains elusive whether the Treg cell reduction observed in *Sanroque* mice with defective ICOS signalling contributes to SLE pathogenesis through Tfh cell-independent non-GC B cell responses.

It has been reported that age-associated B cells (ABCs) are also closely associated with SLE^22^. ABCs can be characterized by the expression of CD11c, CD11b and Tbet. These cells serve as age- or sex-related risk factors, contributing to the pathogenesis of autoimmune diseases, including SLE^23,24^. Interestingly, ABCs primarily arise outside of GCs through a non-GC B cell activation pathway^3^. In this context, PD-1^hi^ CXCR5^−^ CD4^+^ T cells, known as peripheral helper T (Tph) cells, rather than PD-1^hi^ CXCR5^+^ Tfh cells, play a crucial role in the induction of ABCs^25,26^. Thus, it is plausible that the ICOS deficiency and the subsequent reduction of Treg cells are closely associated with the augmentation of ABCs in a Tfh cell-independent manner. Notably, a subset of CD4^+^ conventional T cells (CD4^+^ Tconv) express neuropilin-1 (Nrp-1)^27^, a cell surface molecule predominantly expressed in thymus-origin Treg cells^28,29^. These Nrp-1^hi^ CD4^+^ Tconv cells increase in a SLE disease model and SLE patients, and represent self-reactive effector T cells^27^. However, it remains elusive whether Nrp-1^hi^ CD4^+^ Tconv cells can mediate the production of autoreactive antibodies in SLE by promoting GC or non-GC B cell responses.

In this study, we investigate the underlying mechanisms responsible for the increase in Treg cells in *Sanroque* mice. *Sanroque* Treg cells retain suppressive functions without producing proinflammatory cytokines. Augmentation of Treg cells in *Sanroque* mice is mainly caused by CD69^+^ Treg cell expansion in an ICOS signalling-dependent manner. Conversely, defective ICOS signalling leads to a significant reduction in Treg cell numbers and the concomitant increase in IFN-γ-producing autoreactive CD4^+^ T cells, which are associated with Nrp-1 expression. The Nrp-1^hi^ CD4^+^ Tconv cells can accumulate even in the presence of Treg cells through competitive engagement with dendritic cells (DCs). These cells can act as Tph cells and contribute to the augmentation of ABCs, which potentially drive the production of autoreactive antibodies even in the absence of Tfh cells. These results also suggest that blocking ICOS signalling to prevent the induction of Tfh cells in SLE may inadvertently promote T cell-mediated inflammation and non-GC B cell responses through the induction of ABCs.

## Materials and methods

### Mice

All mice were maintained in the animal facility of POSTECH Biotech Center (South Korea) under specific pathogen-free (SPF) conditions. C57BL/6 *(*B6*)*, *Rag1*^−^^/−^, *Icosl*^−/−^ and OT-II mice (*C57BL/6-Tg(TcraTcrb)425Cbn/J)* were purchased from The Jackson Laboratory. *Sanroque* (*Rc3h1*^San^) and CD45.1 *Foxp3*^IRES-GFP (GFP)^ mice were kindly provided by Vinuesa, C.G. (Australian National University) and Alexander Rudensky (Sloan Kettering Institute), respectively. *Icosl*^−/−^ *Sanroque* (*Icosl*^−/−^ *Rc3h1*^San^) and *Icosl*^−/−^ *Rag1*^−/−^ mice were generated by crossing *Icosl*^−/−^ mice with *Sanroque* and *Rag1*^−/−^ mice, respectively. CD45.1 *Foxp3*^GFP^ mice were crossed with B6 and *Sanroque* mice to generate CD45.1/CD45.2 *Foxp3*^GFP^ and CD45.2 *Sanroque Foxp3*^GFP^ mice. All experiments were conducted using age-matched female mice unless specified otherwise. Germ-free (GF) mice were maintained in sterile isolators (Class Biological Clean Ltd.). Antigen-free (AF) mice were generated by feeding GF mice with a special diet devoid of antigenic macromolecules such as proteins and polysaccharides^30^. All animal experiments were approved and performed in accordance with ethical guidelines of the Institutional Animal Care and Use Committee of POSTECH (IACUC #POSTECH-2020-0067).

### Mixed bone marrow chimera

For generating mixed bone marrow chimera (BMC), 7–8-week-old *Rag1*^−/−^ mice were irradiated at a dose of 6 Gy (600 rad), and the next day, intravenously injected with a total of 1×10^7^ T cell-depleted bone marrow (BM) cells. T cell-depleted BM cells with WT (CD45.1) and *Sanroque* (CD45.2) origin, from age-matched mice, were mixed in a 1:1 ratio. As a control, irradiated *Rag1*^−/−^ mice were injected with 1:1 mixture of CD45.1 and CD45.2 WT B6 T cell-depleted BM cells. The BMC mice were examined 8 weeks post-BM reconstitution.

### Cell isolation

Spleens, mesenteric lymph nodes and peripheral lymph nodes were harvested from various strains of mice. Peripheral lymph nodes included superficial cervical, axillary, brachial and inguinal nodes. Single-cell suspensions were prepared by mechanical dissociation in DMEM supplemented with 1% fetal bovine serum (FBS; Cytiva, USA), 100 U/ml penicillin (Gibco, USA) and 100 µg/ml streptomycin (Gibco). For spleen preparation, RBCs were lysed with ACK lysing buffer (Gibco). For fluorescence-activated cell sorting (FACS) of T and B cell subsets, splenocytes and lymph-node cells were resuspended in DPBS supplemented with 2% FBS, 100 U/ml penicillin and 100 µg/ml streptomycin (Gibco) and sorted using a MoFlo Astrios cell sorter (Beckman Coulter).

### Flow cytometric analysis

Dead cells were excluded by staining Ghost viability dye (Tonbo Biosciences). Fluorochrome-labelled or streptavidin-conjugated antibodies specific for the following surface and intracellular markers were used: B220 (RA3-6B2), CD11b (M1/70), CD11c (N418), CD19 (1D3), CD21/35 (7E9), CD23 (B3B4), CD25 (PC61), CD27 (LG.3A10), CD38 (90), CD4 (RM4-5), CD44 (IM7), CD45.1 (A20), CD45.2 (104), CD62L (MEL-14), CD69 (H1.2F3), CD95 (JO2), CTLA-4 (UC10-4B9), Foxp3 (FJK-16s), GL-7 (GL7), ICOS (C398.4 A), IFN-γ (XMG1.2), IgD (11-26 c.2a), Ki67 (16A8), Ly6C (HK1.4), Ly6G (RB6-8C5), Nrp-1 (FAB566A), OX-40 (OX-86), PD-1 (RMP1-30), Sca-1 (D7), Tbet (O4-46), TCR-β (H57-597), Thy1.1 (OX-7) and Thy1.2 (1953.2.1). Antibodies were purchased from Thermo Fisher Scientific, TONBO Biosciences, BioLegend, R&D Systems and BD Biosciences. For surface staining, cells were stained in FACS buffer (DPBS supplemented with 1% FBS, 100 U/ml penicillin and 100 µg/ml streptomycin). For intracellular staining, surface-stained cells were fixed and permeabilized using Foxp3/Transcription factor staining buffer set (Thermo Fisher Scientific), and stained with fluorochrome-labelled antibodies according to the manufacturer’s instructions. For intracellular cytokine analysis, isolated cells were stimulated for 3 h at 37°C with a cell stimulation cocktail containing protein transport inhibitors (Thermo Fisher Scientific) in complete RPMI-1640 medium (Welgene Korea) supplemented with 10% FBS (Cytiva), 100 U/ml penicillin and 100 μg/ml streptomycin (Gibco), 1x Non-Essential Amino Acids (Welgene), 2 mM L-glutamine (Sigma-Aldrich, USA), 25 mM HEPES (Welgene) and 50 μM β-mercaptoethanol (Sigma-Aldrich). After stimulation, cells were collected in FACS buffer and stained with appropriate antibody panels. Data were acquired using CytoFLEX (Beckman Coulter) at the POSTECH Microbiome Core Facility and analysed using FlowJo software v10 (Tree Star Inc).

### In vivo Treg cell suppression assay and Treg cell stability test

Naïve CD4^+^ Tconv cells and Treg cells were FACS-sorted from spleen, peripheral and mesenteric lymph nodes of *Foxp3*^GFP^ mice carrying congenic markers (CD45.1, CD45.1/CD45.2) or CD45.2 *Sanroque Foxp3*^GFP^ mice to enable donor-specific discrimination after adoptive transfer. To enrich for CD4^+^ T cells, spleen and lymph-node cells were first depleted of non-CD4^+^ cell populations using biotinylated monoclonal antibodies (mAbs) against B220 (RA3-6B2), CD8α (53-6.7), CD11b (M1/70), CD11c (N418), CD19 (MB19-1), CD24 (M1/69) and NK1.1 (PK136), followed by magnetic separation (IMag, BD Biosciences). Dead cells were excluded using propidium iodide (500 ng/ml per 1 × 10^6^ cells; Sigma-Aldrich). Cells were stained with fluorochrome-labelled antibodies for TCR-β, CD4, CD44 and CD62L. Naïve CD4^+^ T cells (PI^–^ CD4^+^ TCR-β^+^ GFP^–^ CD44^lo^ CD62L^hi^) and Treg cells (PI^–^ CD4^+^ TCR-β^+^ GFP^+^) were sorted using a MoFlo Astrios cell sorter (Beckman Coulter). For the in vivo Treg suppression assay, 3 × 10⁵ naïve CD4^+^ Tconv cells (from CD45.1 *Foxp3*^GFP^ mice) and 1.5 × 10⁵ Treg cells were co-transferred into *Rag1*^−/−^ mice. Treg cells were isolated from CD45.1/CD45.2 *Foxp3*^GFP^ or CD45.2 *Sanroque Foxp3*^GFP^ mice. Analysis was performed 4 weeks after adoptive transfer. For the Treg maintenance or stability test, 5 × 10⁵ naïve CD4⁺ Tconv cells (from CD45.1 *Foxp3*^GFP^ mice) and 1.25 × 10⁵ Treg cells from either CD45.1/CD45.2 Foxp3^GFP^ mice or *Sanroque* CD45.2 *Foxp3*^GFP^ mice were co-transferred into either *Rag1*^−/−^ or *Icosl*^−/−^ *Rag1*^−/−^ mice. Treg stability was assessed 6 weeks post-transfer by analysing the persistence and phenotype of GFP^+^ CD4^+^ T cells from each donor population.

### Enzyme-linked immunosorbent assay

Levels of IgG in serum were analysed by enzyme-linked immunosorbent assay (ELISA). ELISA plates (Costar, USA) were coated with 2 μg/ml purified anti-mouse IgG (Southern Biotech, Cat. # 1010-01) overnight at 4°C. Plates were then blocked with PBS containing 0.1% Tween-20 and 3% skimmed milk for 1 h at room temperature (RT). Diluted serum samples and reference IgG standards (Southern Biotech, Cat. #0107-01) were added and incubated for 2 h at RT. After washing, HRP-conjugated anti-mouse IgG (Southern Biotech, Cat. #1036-05) was added and incubated for 1 h at RT. The peroxidase reaction was visualised by adding TMB substrate solution (Sigma-Aldrich). Plates were read at 450 nm using a spectrophotometer (Tecan Infinite 200 microplate reader).

### Antinuclear antibody test

Levels of anti-dsDNA IgG in serum were analysed by antinuclear antibody (ANA) test. ELISA plates were coated with 0.01% Poly-L-Lysine solution (Sigma) for 5–10 min at 37°C. Then, plates were coated with 10 µg/ml Calf Thymus DNA (Trevigen) overnight at 4°C. After incubation, plates were blocked with blocking buffer (10% FBS in PBS) for 2 h at RT and diluted serum samples were added. After a 2-h incubation, HRP-conjugated anti-mouse IgG was added. After incubation for 1 h, peroxidase reaction was visualised by adding TMB substrate solution. Plates were read at 450 nm using a spectrophotometer.

### Histopathological examination

Lung, liver and kidney tissues were collected following PBS perfusion. Tissues were fixed in 4% paraformaldehyde (PFA) solution, embedded in paraffin and sectioned at a thickness of 5 µm. Lung and liver sections were stained with haematoxylin and eosin (H&E), and kidney sections were stained with periodic acid–Schiff (PAS) according to standard protocols. Lung pathology was assessed based on alveolar septal thickening (0–3), airway collagen deposition (0–3), immune infiltration in the alveolar and interstitial spaces (0–3) and alveolar distortion (0–3). Liver pathology was evaluated by scoring cholestasis (0–3), inflammation (0–3), fibrosis (0–3) and bile-duct proliferation (0–3). Kidney pathology was scored by glomerular mesangial proliferation (0–3), necrotic tubules (0–3) and inflammatory cell infiltration (0–3). Images were acquired using the Axioscan7 microscope slide scanner, and analysed by ZEN microscopy software (Zeiss).

### Confocal imaging of dendritic cell–T cell interactions

Glass-bottomed dishes (35 mm; Cellvis) were coated overnight at 4°C with bovine fibronectin (25 µg/ml; Sigma-Aldrich) and rinsed twice with PBS. Splenocytes were isolated from *Foxp3*^GFP^ mice or OT-II mice, and DCs (MHC-II^hi^ CD11c^+^), Treg cells (CD4^+^ TCR-β^+^ GFP^+^ from *Foxp3*^GFP^ mice or CD4^+^ TCR-β^+^ CD25^+^ from OT-II mice), Nrp-1^lo^ and Nrp-1^hi^ CD44^hi^ CD4^+^ Tconv cells were FACS sorted. Immediately after sorting, cells were labelled with DiD (far-red), DiO (green) or DiI (orange/red) (Thermo Fisher Scientific) in serum-free RPMI-1640 (5 µl per 10^6^ cells) for 20 min at 37°C, washed twice, and cultured in phenol red–free complete RPMI-1640 (10% FBS, 100 U/ml penicillin, 100 µg/ml streptomycin, 2 mM L-glutamine, 50 µM β-mercaptoethanol). Labelled cells were seeded into coated dishes at 2 × 10^4^ DCs, 1 × 10^5^ Treg cells, and either 1 × 10^5^ Nrp-1^lo^ or Nrp-1^hi^ CD44^hi^ CD4^+^ Tconv cells per dish. Cells from *Foxp3*^GFP^ mice were stimulated with 1 μg/ml purified anti-CD3ε (145-2C11, TONBO Biosciences), whereas cells from OT-II mice were stimulated with 10 μg/ml OVA₃₂₃₋₃₃₉ peptide (AnaSpec). After 24-h incubation at 37°C with 5% CO_2_, live cell imaging was performed using a Carl Zeiss Confocal LSM900 (×20 objective). To compare DC–T cell interaction between OT-II cells and DCs at 12 or 24 h after seeding in the absence or presence of OVA₃₂₃₋₃₃₉ peptide, cells were prepared and seeded under the same conditions as described above. Cultures were fixed with 4% PFA at each time point. Following fixation, PFA was gently aspirated and replaced with PBS to preserve cell–substrate interactions and allow imaging of adherent cells on the dish surface. Image processing was carried out in ImageJ/Fiji, and quantitative analysis of cell interactions was performed using custom Python scripts (v3.9). For quantification of DC–T cell interactions, individual cell masks were generated for DCs, Treg cells and either Nrp-1^lo^ or Nrp-1^hi^ CD44^hi^ CD4^+^ Tconv cells. To define physical contact, DC masks were computationally expanded by approximately 2 µm (7 pixels; pixel size = 0.312 µm) to approximate the cell-membrane boundary. Treg or Tconv cells within this expanded boundary were classified as interacting cells based on mask overlap. The number of interacting cells was then quantified for each field of view using custom Python scripts. Z-stack images were additionally acquired at 1-µm intervals across the central region of the cells using a ×20 objective to enable three-dimensional validation of cell–cell contact. Contact events defined by mask overlap in 2D analyses were confirmed by examining membrane overlap or direct membrane proximity between DCs and T cells across adjacent z-planes.

### In vitro T cell proliferation assay

Splenocytes and lymph-node cells were isolated from CD45.1 *Foxp3*^GFP^ mice. DCs (MHC-II^hi^ CD11c⁺), Treg cells (CD4⁺ TCR-β^+^ GFP⁺) and Nrp-1^lo^ and Nrp-1^hi^ CD44 ^hi^ CD4^+^ Tconv (CD4⁺ TCR-β^+^ GFP^–^) cells were FACS sorted. Immediately after sorting, Nrp-1^lo^ and Nrp-1^hi^ CD44 ^hi^ CD4^+^ Tconv cells were labelled with CellTrace™ Violet (CTV, Invitrogen) according to the manufacturer’s instructions. A total of 2.5 × 10⁴ Tconv cells were co-cultured with 1.25 × 10⁴ DCs in the presence or absence of Treg cells at Treg:Tconv ratios of 1:2, 1:5 or 1:10 in complete RPMI-1640 medium in 96-well flat-bottomed plates (Corning, USA). Cells were stimulated with soluble 1 µg/ml purified anti-CD3ε antibody (145-2C11, Tonbo Biosciences) and incubated for 3 days at 37°C with 5% CO₂. T cell proliferation was assessed by CTV dilution using flow cytometry.

### In vitro age-associated B cell differentiation

CD23⁺ CD21^int^ follicular B (FoB) cells were FACS sorted from the spleens of 6–7-week-old WT B6 mice using a MoFlo Astrios cell sorter, and then cultured in flat-bottomed 24-well plates (Corning, USA) in 1 ml of complete RPMI-1640 medium (Welgene) supplemented with 10% FBS (Cytiva), 100 U/ml penicillin and 100 µg/ml streptomycin (Gibco), 1× non-essential amino acids (Welgene), 2 mM L-glutamine (Sigma-Aldrich), 25 mM HEPES (Welgene), and 50 µM β-mercaptoethanol (Sigma-Aldrich). As a negative control, 1 × 10⁶ FoB cells were stimulated with 5 µg/ml F(ab’)₂ anti-mouse IgM (Jackson ImmunoResearch, 115-006-075) and 5 µg/ml Ultra-LEAF™ (Low Endotoxin, Azide-Free) purified anti-mouse CD40 (FGK45, BioLegend). As a positive control for ABC differentiation, FoB cells were stimulated with anti-mouse IgM, and anti-CD40 antibodies in the presence of an ABC-inducing cocktail (ABC cocktail) consisting of 1 µg/ml of imiquimod (InvivoGen, USA), 50 ng/ml of murine recombinant IL-21 (Peprotech, USA), 20 ng/ml of murine recombinant IFN-γ (Peprotech, USA). For T cell co-culture experiments, Nrp-1^lo^ or Nrp-1^hi^ CD44^hi^ CD4⁺ Tconv cells from WT B6 or *Sanroque* mice were FACS sorted, and 5 × 10⁵ CD4⁺ T cells were added to FoB cells in flat-bottomed 24-well plates (Corning) for 3 days in 1 ml of complete RPMI-1640 medium per well. Wells were pre-coated with 1 µg/ml purified anti-CD3ε (145-2C11, Tonbo Biosciences) and 2 µg/ml purified anti-CD28 (37.51, Tonbo Biosciences) antibodies. To inhibit inducible T cell costimulator ligand (ICOSL) signalling, anti-ICOSL antibodies (HK5.3, Bio X Cell) were added to the co-culture at 10 µg/ml. Cells were cultured for 3 days before harvesting.

### Transwell co-culture assay

To examine whether cell-to-cell contact is required for the enhancement of ABC induction by Nrp-1^hi^ CD44 ^hi^ CD4^+^ Tconv cells, transwell co-culture experiments were performed using 24-well transwell plates (0.4 µm pore size; Corning, USA). Nrp-1^hi^ CD44^hi^ CD4⁺ Tconv cells were seeded in the lower chamber, and CD23⁺ CD21^int^ FoB cells were placed in the upper transwell insert. Cell numbers and culture conditions were identical to those used in the co-culture experiments described above. For T cell activation, the lower wells were pre-coated with 1 µg/ml purified anti-CD3ε (145-2C11, Tonbo Biosciences) and 2 µg/ml purified anti-CD28 (37.51, Tonbo Biosciences) mAbs prior to seeding T cells. Cells were cultured for 3 days in complete RPMI-1640 medium and harvested for analysis.

### Immunofluorescence staining

Spleens were isolated from WT B6, *Sanroque* and *Icosl*^−/−^ *Sanroque* mice, fixed in 4% PFA solution, and cryoprotected in 30% sucrose until fully equilibrated. Tissues were embedded in O.C.T. compound (Sakura Tissue-Tek) and frozen. Frozen blocks were cryo-sectioned at a thickness of 7 μm, followed by fixation in 2% PFA for 15 min. Blocking was performed for 2 h at room temperature using PBS containing 5% BSA and Fc blocker (1:200; BD Biosciences). Normal rat serum (1% v/v; Invitrogen) or normal goat serum (1% v/v; Invitrogen) was included in the blocking buffer as appropriate for subsequent secondary antibodies. For visualization of T cell and B cell zones, sections were incubated overnight at 4°C with biotin-conjugated anti-mouse B220 (1:100; BD Biosciences), followed by incubation with streptavidin-FITC (1:250; BioLegend) and anti-mouse CD4-PE (1:100; BioLegend). To visualize Nrp-1⁺ CD4⁺ T conventional cells relative to B cell follicles, sections were incubated overnight at 4°C with biotin-conjugated anti-mouse Foxp3 (1:100; Invitrogen), and rabbit anti-mouse Nrp-1 (1:100; Invitrogen), followed by streptavidin–Alexa Fluor 488 (1:250; Invitrogen) and goat anti-rabbit Alexa Fluor 568 (1:1000; Invitrogen), and anti-mouse B220-APC (1:100; BioLegend). To confirm CD4 expression on Nrp-1⁺ cells and their proximity to B cells, sections were incubated overnight at 4°C with rat anti-mouse Nrp-1 (1:100; Invitrogen) and biotin-conjugated anti-mouse B220 (1:100; BD Biosciences), followed by goat anti-rat Alexa Fluor 488 (1:1000; Invitrogen), streptavidin–Alexa Fluor 647 (1:250; Invitrogen) and anti-mouse CD4-PE (1:100; BioLegend). All sections were counterstained with DAPI (1:1000; Sigma-Aldrich) to visualize nuclei. Images were acquired using a Leica DM6B fluorescence microscope.

### Statistical analysis

Statistical analysis was performed by using Prism 6.0 (GraphPad Software). All data were presented as mean ± standard error of the mean (SEM). Statistical significance was analysed using unpaired or paired Student’s *t* test and one-way or two-way analysis of variance with Tukey’s multiple comparisons test. A *P* value < 0.05 was considered statistically significant (*P < 0.05; **P < 0.01, ***P < 0.001; ns, not significant).

## Results

### Treg cells expand during the onset of systemic lupus erythematosus-like disease and retain suppressive function in *Sanroque* mice

**Fig. 1:**
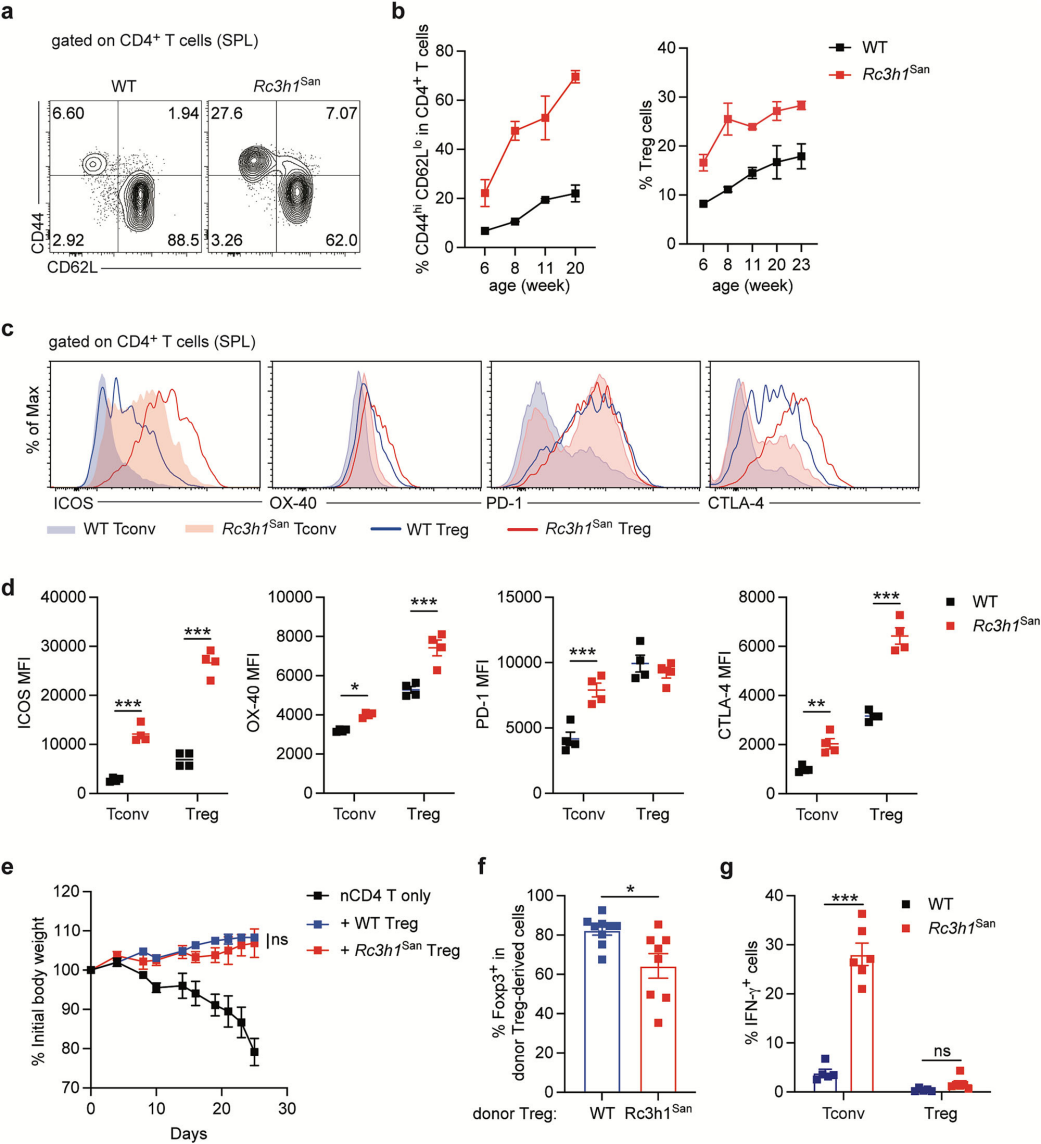
Foxp3^+^ regulatory CD4^+^ T cells with intact suppressive activity increase during the onset of systemic lupus erythematosus-like disease in *Sanroque* mice. **a**, **b** Single-cell suspensions were prepared from spleen (SPL) from wild type (WT) and *Sanroque* mice (*Rc3h1*^San^) of the indicated ages. **a** Representative contour plots of CD44 and CD62L gated on splenic Foxp3^−^ CD4^+^ conventional T (Tconv) cells from 8-week-old WT and *Sanroque* mice. **b** Frequency of effector/memory-phenotype (CD44^hi^ CD62L^lo^) Tconv cells gated on CD4^+^ Tconv cells (left) and Treg cells (Foxp3^+^) gated on CD4^+^ T cells (right) in WT and *Sanroque* mice of the indicated ages (*n* = 2 per group at each time point). **c**, **d** Splenocytes from 13-week-old WT and *Sanroque* female mice were prepared to examine surface marker expression on CD4^+^ T cell subsets (*n* = 4 per group). **c** Representative histograms of inducible T cell costimulator (ICOS), OX-40, PD-1 and CTLA-4 in WT and *Sanroque* splenic CD4^+^ T cells depending on Foxp3 expression. **d** Median fluorescence intensity (MFI) of indicated cell surface markers in CD4^+^ Tconv cells and Treg cells in the spleen from WT and *Sanroque* mice. Two independent experiments show similar results. **e**, **f** To examine in vivo suppressive function of WT and *Sanroque* Treg cells, either Thy1.1 WT Treg cells or CD45.2^+^ *Sanroque* Treg cells were co-transferred into *Rag1*^−/−^ mice with CD45.1^+^ naïve CD4^+^ Tconv cells. **e** Initial body weight changes after adoptive transfer are shown. At least two independent experiments show similar results. **f** Frequency of Foxp3^+^ Treg cells in donor WT Treg cells and *Sanroque* Treg cells at day 28 after adoptive transfer. Data are pooled from two independent experiments (*n* = 9 for WT Treg cells, *n* = 8 for Sanroque Treg cells). **g** Frequency of IFN-γ^+^ cells gated on CD4^+^ T cells in the spleen of WT and *Sanroque* mice (*n* = 5 for WT, *n* = 6 for *Sanroque* mice). Two independent experiments show similar results. Statistical differences were determined by two-way analysis of variance with Bonferroni multiple comparisons test (**d**) or one-way analysis of variance with Tukey’s multiple comparisons test (e) and unpaired two-tailed Student’s *t* test (**f,** **g**). *P < 0.05, **P < 0.01, ***P < 0.001. ns, not significant. Error bars represent SEM. Each symbol represents an individual mouse.

To investigate the fate of Treg cells during the onset of SLE-like disease in *Sanroque (Rc3h1*^San^*)* mice, we examined the proportions of CD44^hi^ CD62L^lo^ CD4^+^ Tconv cells and Treg cells depending on age. By 6 weeks of age, female *Sanroque* mice exhibited markedly elevated levels of CD4^+^ Tconv cells in peripheral lymphoid organs compared with wildtype (WT) controls (Fig. 1a, b). CD4^+^ Tconv cells progressively increased with age in parallel with disease progression (Fig. 1b). Notably, both the frequency and absolute number of Treg cells were significantly increased in *Sanroque* mice relative to WT controls across all examined lymphoid organs (Supplementary Fig. 1a). The proportion of Treg cells expressing Ki67, a molecule expressed during cell-cycle progression, was approximately two-fold higher in *Sanroque* mice relative to Treg cells in WT controls (Supplementary Fig. 1b, c). These results indicate that Treg cells in *Sanroque* mice accumulate during the onset of SLE-like disease through peripheral proliferation.

Next, to examine whether these expanded Treg cells in *Sanroque* mice retain suppressive functions, we first examined the surface expression of the key immune-regulatory molecules. ICOS and OX-40 mRNAs are targets of Roquin proteins^7^. The expression of these molecules was significantly increased in both CD4^+^ Tconv cells and Treg cells in *Sanroque* mice compared with those in WT mice (Fig. 1c, d). Interestingly, Treg cells in *Sanroque* mice expressed higher levels of ICOS than CD4^+^ Tconv cells (Fig. 1c, d). Although PD-1 expression markedly increased in CD4^+^ Tconv cells in *Sanroque* mice, PD-1 expression in *Sanroque* Treg cells was comparable with that of WT Treg cells. In contrast, CTLA-4 expression was significantly elevated in *Sanroque* Treg cells compared with WT Treg cells, suggesting that *Sanroque* Treg cells might exhibit greater suppressive activity than WT Treg cells.

To directly assess the suppressive function of Treg cells in vivo, we performed an in vivo Treg suppression assay using co-transfer of either WT or *Sanroque* Treg cells with WT naïve CD4^+^ Tconv cells into lymphopenic *Rag1*^−/−^ mice. Each donor T cell population was distinguished by congenic markers such as Thy1.1 or CD45.1. *Sanroque* Treg cells effectively suppressed colitis-induced body weight loss to a similar extent as WT Treg cells, demonstrating functional competence in vivo (Fig. 1e). As reported in the previous study^31^, WT Treg cells co-transferred with naïve CD4^+^ Tconv cells were highly stable not to lose Foxp3 expression. Interestingly, a considerable proportion of *Sanroque* Treg cells lost Foxp3 expression and became ‘ex-Treg cells’, though this did not impair their overall suppressive activity (Fig. 1f).

Treg cells can produce pro-inflammatory cytokines under inflammatory conditions^20,32,33^. As reported previously^6^, IFN-γ production in CD4^+^ Tconv cells was markedly increased in *Sanroque* mice (Fig. 1g). Despite a notable increase in IFN-γ-producing CD4^+^ Tconv cells, Treg cells in *Sanroque* mice did not produce IFN-γ (Fig. 1g). Overall, these results suggest that Treg cells with intact suppressive functions accumulate during the onset of SLE-like disease in *Sanroque* mice.

### Treg expansion in *Sanroque* mice results from T cell-intrinsic mechanisms rather than environmental cytokine availability

The parallel expansion of Treg cells and CD4^+^ Tconv cells in *Sanroque* mice raised the question of whether Treg cell accumulation results from cell-intrinsic changes or arises from alterations in the shared immune environment. Interleukin-2 (IL-2) promotes the proliferation and maintenance of Treg cells and IL-2 signalling is also required for the suppressive function of Treg cells^34,35^. In this regard, IL-2, mostly produced by effector CD4^+^ Tconv cells^36,37^, can drive the expansion of Treg cells in *Sanroque* mice. To assess whether IL-2 or other extrinsic cues contribute to Treg cell expansion in *Sanroque* mice, we established mixed bone marrow (BM) chimeras, where WT and *Sanroque* Treg cells were placed in the same microenvironment (Supplementary Fig. 2a). In mixed BM chimeras, we observed a distinct difference in the proportion of Treg cells derived from WT and *Sanroque* BM cells. Notably, the proportion of Treg cells generated from *Sanroque* BM cells was significantly higher than their WT-derived counterparts within the same host (Supplementary Fig. 2b), indicating a T cell-intrinsic propensity for Treg cell accumulation. In contrast, the levels of Treg cells in BM chimeras reconstituted with WT (CD45.1) and WT (CD45.2) BM cells were comparable, thereby excluding technical or host-derived bias (Supplementary Fig. 2c).

Mutated Roquin-1 in *Sanroque* mice results in the increased stability of IFN-γ mRNA^6^. In this regard, we observed an enhanced production of IFN-γ from *Sanroque* CD4^+^ T cells when compared with WT CD4^+^ T cells in mixed BM chimeras, also confirming that effector functions are similarly governed by T cell-intrinsic mechanisms (Supplementary Fig. 2d, e). Collectively, these results suggest that both the expansion of Treg cells and the elevated production of effector cytokines in *Sanroque* mice arise from T cell-intrinsic mechanisms, independent of IL-2 availability or other environmental influences.

### Augmentation of Treg cells in *Sanroque* mice is mainly mediated by inducible T cell costimulator signalling-dependent induction of CD69^+^ Treg cells

Previous studies have shown that ICOS signalling plays a crucial role in promoting Treg cell generation, proliferation and survival, as well as enhancing the suppressive function of Treg cells by promoting IL-10 production^15,16^. Our results are in line with these previous studies. To examine whether the increased Treg cell population in *Sanroque* mice could be attributed to the heightened ICOS signalling in these cells, we first generated *Icosl*^−/−^ *Rag1* ^−/−^ mice and adoptively transferred *Sanroque* Treg cells together with naïve CD4^+^ Tconv cells from WT mice (Supplementary Fig. 3a). Although the co-transfer of *Sanroque* Treg cells effectively prevented the colitis induction, *Sanroque* Treg cells in *Icosl*^−/−^ *Rag1*^−/−^ mice co-transferred with naïve CD4^+^ Tconv cells failed to prevent the colitis-associated body weight loss (Supplementary Fig. 3b). Examination of donor Treg cell population revealed that in *Icosl*^−/−^ *Rag1* ^−/−^ mice, the percentage and number of donor Treg cells were profoundly reduced (Supplementary Fig. 3c, d). However, ICOS dependency on Treg cell maintenance was not unique to *Sanroque* Treg cells with increased ICOS expression because WT Treg cells adoptively transferred into *Icosl*^−/−^ *Rag1* ^−/−^ mice were also significantly reduced and failed to prevent the colitis induction in these recipient mice (Supplementary Fig. 3e–g). These results suggest that ICOS signalling itself is required for the maintenance of Treg cells in inflammatory settings.

**Fig. 2:**
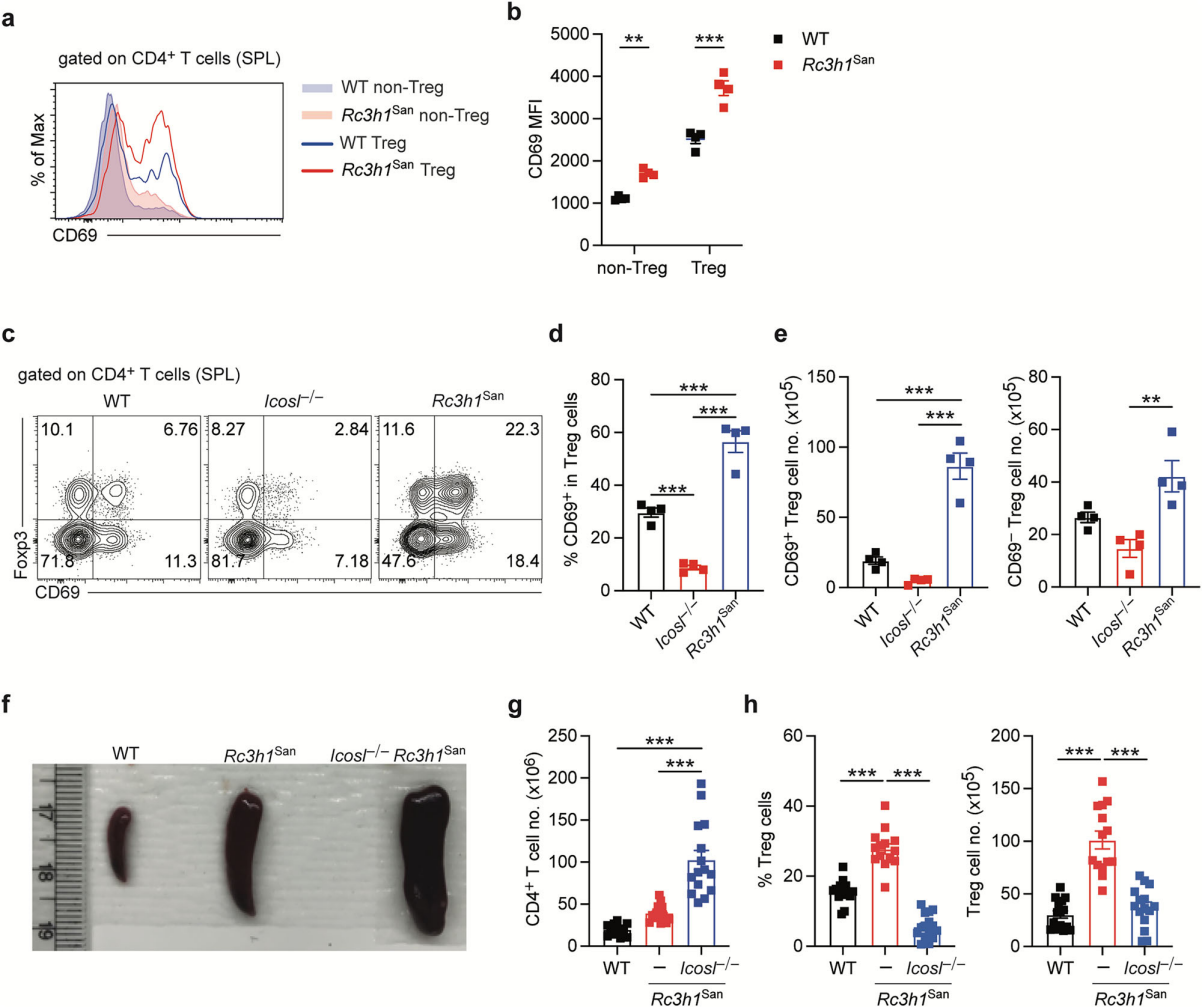
Augmentation of Treg cells in *Sanroque* mice is mainly mediated by inducible T cell costimulator signalling-dependent induction of CD69^+^ Treg cells. **a** Representative histograms showing CD69 expression on splenic CD4^+^ T cells from 13-week-old wild type (WT) and *Sanroque* (*Rc3h1*^San^) mice. **b** Median fluorescence intensity (MFI) of CD69 expression in CD4^+^ Tconv cells and Treg cells in the spleen from WT and *Sanroque* mice (*n* = 4 per group). **c** Representative contour plots of Foxp3 and CD69 gated on splenic CD4^+^ T cells in the indicated mice. **d** Frequency of CD69^+^ Treg cells in total CD4^+^ T cells (*n* = 4 per group). **e** Total number of CD69^+^ (left) and CD69^− ^(right) Treg cells in the spleen in the indicated mice. Two independent experiments show similar results. **f–h** Splenocytes were prepared from 16-week-old WT, *Sanroque* and ICOSL-deficient *Sanroque* (*Icosl*^−/−^ *Rc3h1*^San^) mice. **f** Representative images of spleen from the indicated mice. **g** Total number of CD4^+^ T cells in the spleen. **h** Frequency of Treg cells in CD4^+^ T cells (left) and total number of Treg cells (right) in the spleen from the indicated mice. Data are pooled from six independent experiments (*n* = 16 per WT and *Sanroque*, and *n* = 15 per ICOSL-deficient *Sanroque* mice). Statistical differences were determined by two-way analysis of variance with Bonferroni multiple comparisons test (**b**) or one-way analysis of variance with Tukey’s multiple comparisons test (**d**, **e**, **g,** **h**). **P < 0.01, ***P < 0.001. Error bars represent SEM. Each symbol represents an individual mouse.

Next, we sought to examine phenotypic changes in Treg cells that are associated with the increased ICOS signalling. It has previously been shown that increased ICOS expression in Treg cells is associated with elevated CD69 expression^38^. CD69^+^ Treg cells express higher levels of suppressive mediators such as CTLA-4 and exhibit enhanced suppressive functions compared with CD69^−^ Treg cells^39^. Consistent with these findings, we observed increased CD69 expression in *Sanroque* Treg cells compared with WT Treg cells (Fig. 2a, b). The proportion of CD69^+^ Treg cells was higher in *Sanroque* mice than in WT mice (Fig. 2c, d). Conversely, ICOSL-deficient mice exhibited a reduced proportion of CD69^+^ Treg cells compared with WT controls (Fig. 2c, d). The increase in CD69^+^ Treg cell numbers was more pronounced than that of CD69^−^ Treg cells in *Sanroque* mice compared with WT mice (Fig. 2d, e). These results indicate that the induction of CD69^+^ Treg cells in an ICOS signalling-dependent manner is mainly responsible for the overall augmentation of Treg cells in *Sanroque* mice.

Next, to further examine the influence of defective ICOS signalling on the levels of Treg cells in *Sanroque* mice, we generated ICOSL-deficient *Sanroque* mice (*Icosl*^−/−^ *Rc3h1*^San^ mice). These mice exhibited more pronounced splenomegaly (Fig. 2f) and a significant increase in splenic CD4^+^ T cell number compared with *Sanroque* mice (Fig. 2g). As expected, both the proportion of Treg cells within the CD4^+^ T cell compartment and number of Treg cells were markedly reduced in ICOSL-deficient *Sanroque* mice (Fig. 2h). Notably, defective ICOS signalling led to a greater reduction in CD69^+^ Treg cell numbers than those of CD69^−^ Treg cells (Supplementary Fig. 4a, b). These results suggest that the augmentation of the functional Treg cell population, in particular, CD69^+^ Treg cells, in *Sanroque* mice heavily relies on ICOS signalling, even though these Treg cells remain insufficient to fully restrain autoimmunity in this context.

### Defective inducible T cell costimulator signalling does not fully rescue the systemic lupus erythematosus symptoms in *Sanroque* mice

**Fig. 3:**
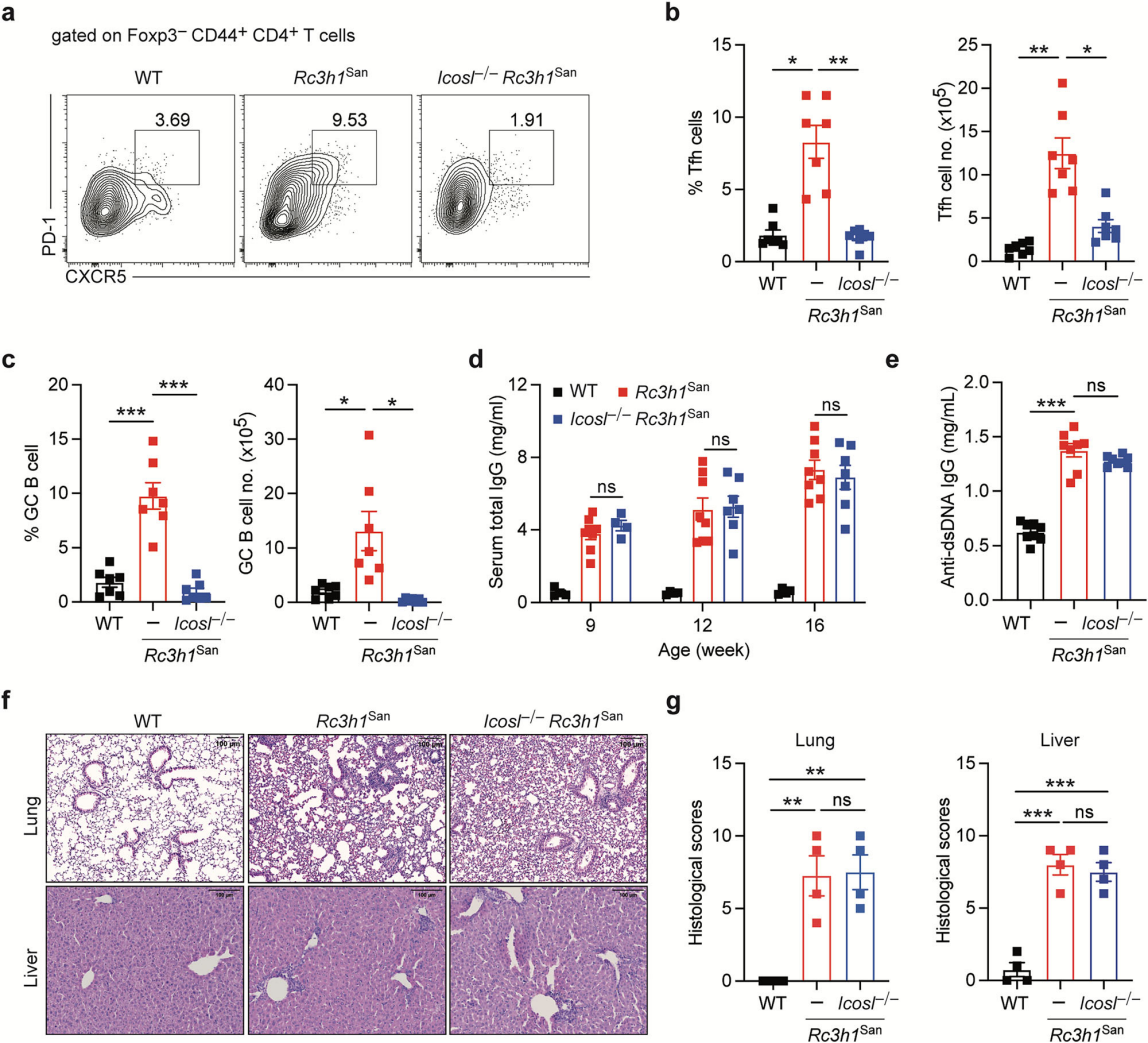
Defective inducible T cell costimulator signalling results in the reduction of Treg cells and does not fully rescue the systemic lupus erythematosus symptoms in *Sanroque* mice. Spleens and splenocytes were prepared from 16-week-old wild type (WT), *Sanroque* (*Rc3h1*^San^) and inducible T cell costimulator ligand (ICOSL)-deficient *Sanroque* (*Icosl*^−/−^ *Rc3h1*^San^) mice. **a** Representative contour plot showing PD-1^hi^ CXCR5^+^ Tfh cells in the indicated mice. **b** Frequency of Tfh cells in Foxp3^−^ CD44^+^ CD4^+^ T cells (left) and total number of Tfh cells (right) in the spleen from the indicated mice. **c** Frequency of GL7^+^ CD95^+^ germinal centre (GC) B cells (left) and total number of GC B cells (right) in the spleen from the indicated mice. Data are pooled from three independent experiments (*n* = 7 per group). **d** Serum IgG levels were determined in WT, *Sanroque* and ICOSL-deficient *Sanroque* mice at the indicated ages (*n* = 4 per WT, *n* = 8 per *Sanroque* and *n* = 7 per ICOSL-deficient *Sanroque* mice). **e** Serum levels of anti-dsDNA specific IgG in 12-week-old WT, *Sanroque* and ICOSL-deficient *Sanroque* mice. Data are pooled from three independent experiments (*n* = 8 per WT and *Sanroque*, and *n* = 7 per ICOSL-deficient *Sanroque* mice). **f** Representative H&E stained images of lung (upper panel) and liver (lower panel). **g** Histological scores in lung (left) and liver (right) (*n* = 4 per group). Statistical differences were determined by one-way analysis of variance with Tukey’s multiple comparisons test (**b**, **c**, **e**, **g**) or two-way analysis of variance with Bonferroni multiple comparisons test (**d**). *P < 0.05, **P < 0.01, ***P < 0.001. ns, not significant. Error bars represent SEM. Each symbol represents an individual mouse.

ICOS–ICOSL interaction is critical for the differentiation of Tfh cells and subsequent induction of GC B cell responses^13^. In line with this, the levels of Tfh cells and GC B cells were significantly reduced in ICOSL-deficient *Sanroque* mice compared with those in *Sanroque* mice (Fig. 3a–c). However, consistent with previous findings^6^, defective ICOS signalling did not completely reverse the SLE-like disease phenotype in *Sanroque* mice. The serum levels of total IgG and autoreactive IgG specific to double-stranded DNA (anti-dsDNA IgG) remained comparable between *Sanroque* and ICOSL-deficient *Sanroque* mice (Fig. 3d, e).

Histological analysis also revealed that inflammation in the lung, liver and kidney of *Sanroque* and ICOSL-deficient *Sanroque* mice was comparable (Fig. 3f, g and Supplementary Fig. 5a, b). These results suggest that SLE pathogenesis in ICOSL-deficient *Sanroque* mice may be driven by mechanisms independent of GC B cell responses, which depend on Tfh cells.

### Defective inducible T cell costimulator ligand signalling disrupts B cell follicle structure and drives a shift toward germinal centre-independent autoimmunity in *Sanroque* mice

**Fig. 4:**
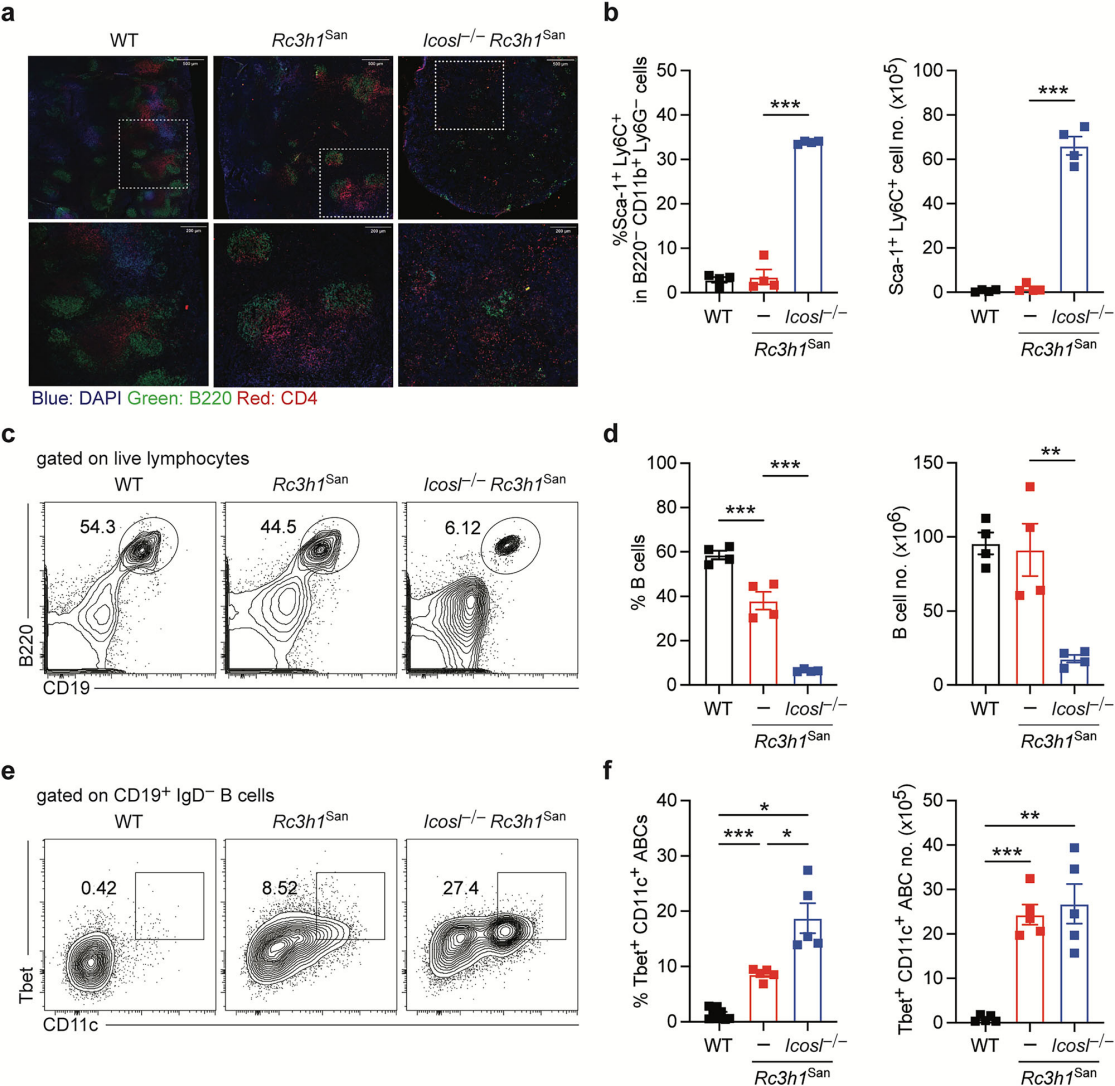
Defective inducible T cell costimulator ligand signalling is associated with disrupted B cell follicle structure and an increase in age-associated B cells in *Sanroque* mice. Spleens from 16-week-old female wild type (WT), *Sanroque* (*Rc3h1*^San^) and inducible T cell costimulator ligand (ICOSL)-deficient *Sanroque* (*Icosl*^−/−^ *Rc3h1*^San^) mice were prepared and sectioned (*n* = 4 per group). **a** Representative immunofluorescence image of the spleen was reconstructed by tiling the images (upper panel) and the splenic B cell follicle and T cell zone are shown (lower panel) as magnified views of the boxed regions in the upper panels. The scale bar indicates 500 μm (upper panel) and 200 μm (lower panel), respectively. DAPI is shown in blue, CD4 in red and B220 in green. **b** Frequency of Sca-1^+^ Ly6C^+^ monocytes gated on B220^−^ CD11b^+^ Ly6G^−^ cells (left) and total number of Sca-1^+^ Ly6C^+^ monocytes (right) in spleen. **c** Representative contour plot showing B220^+^ CD19^+^ B cells gated on live lymphocyte population. **d** Frequency of B220^+^ CD19^+^ B cells gated on live lymphocyte population (left) and total number of B cells (right) in spleen of the indicated mice. Two independent experiments show similar results. **e** Representative contour plot showing Tbet^+^ CD11c^+^ age-associated B cells (ABCs) in the indicated mice. **f** Frequency of Tbet^+^ CD11c^+^ ABCs in CD19^+^ IgD^−^ B cells (left) and total number of ABCs in the spleen from the indicated mice (*n* = 5 per group). Data are pooled from two independent experiments. Statistical differences were determined by one-way analysis of variance with Tukey’s multiple comparisons test (**b**, **d**, **f**). *P < 0.05, **P < 0.01, ***P < 0.001. Error bars represent SEM. Each symbol represents an individual mouse.

We next tried to investigate the underlying mechanisms responsible for autoreactive IgG production in ICOSL-deficient *Sanroque* mice. For this purpose, we first examined lymphoid structures in the spleen of ICOSL-deficient *Sanroque* mice. Immunofluorescence staining of spleen sections revealed that compared with WT mice, *Sanroque* mice also exhibited well-organized lymphoid structures, characterized by B cell follicles with adjacent T cell zones (Fig. 4a). As supported by flow cytometric analysis, CD4^+^ T cells infiltrated into B cell follicles were obviously increased in *Sanroque* mice. Interestingly, these organized lymphoid structures were markedly reduced in ICOSL-deficient *Sanroque* mice (Fig. 4a). As reported previously^40^, Sca-1^+^ Ly6C^+^ monocytes, which can result in GC collapse, were massively increased in the spleen of ICOSL-deficient *Sanroque* mice (Fig. 4b). These results suggest that the non-GC B cell activation pathway presumably mediates the induction of autoreactive IgG in ICOSL-deficient *Sanroque* mice.

As mentioned previously, ABCs are a hallmark of non-GC B cell responses rather than GC B cell responses^3^. Therefore, we examined the presence of ABCs, defined as CD19^+^ IgD^−^ Tbet^+^ CD11c^+^ B cells in mice^41^. Although the levels of B cells were significantly reduced in ICOSL-deficient *Sanroque* mice, compared with *Sanroque* mice (Fig. 4c, d), we found that the proportion of ABCs in B cell populations was significantly elevated in ICOSL-deficient *Sanroque* mice compared with *Sanroque* mice (Fig. 4e, f). These results suggest that ABCs play a key role in mediating autoreactive IgG production in ICOSL-deficient *Sanroque* mice and their induction can occur obviously in the absence of Tfh cells.

### IFN-γ-producing self-reactive Nrp-1^hi^ conventional CD4^+^ T cells accumulated in *Sanroque* and inducible T cell costimulator ligand-deficient *Sanroque* mice

**Fig. 5:**
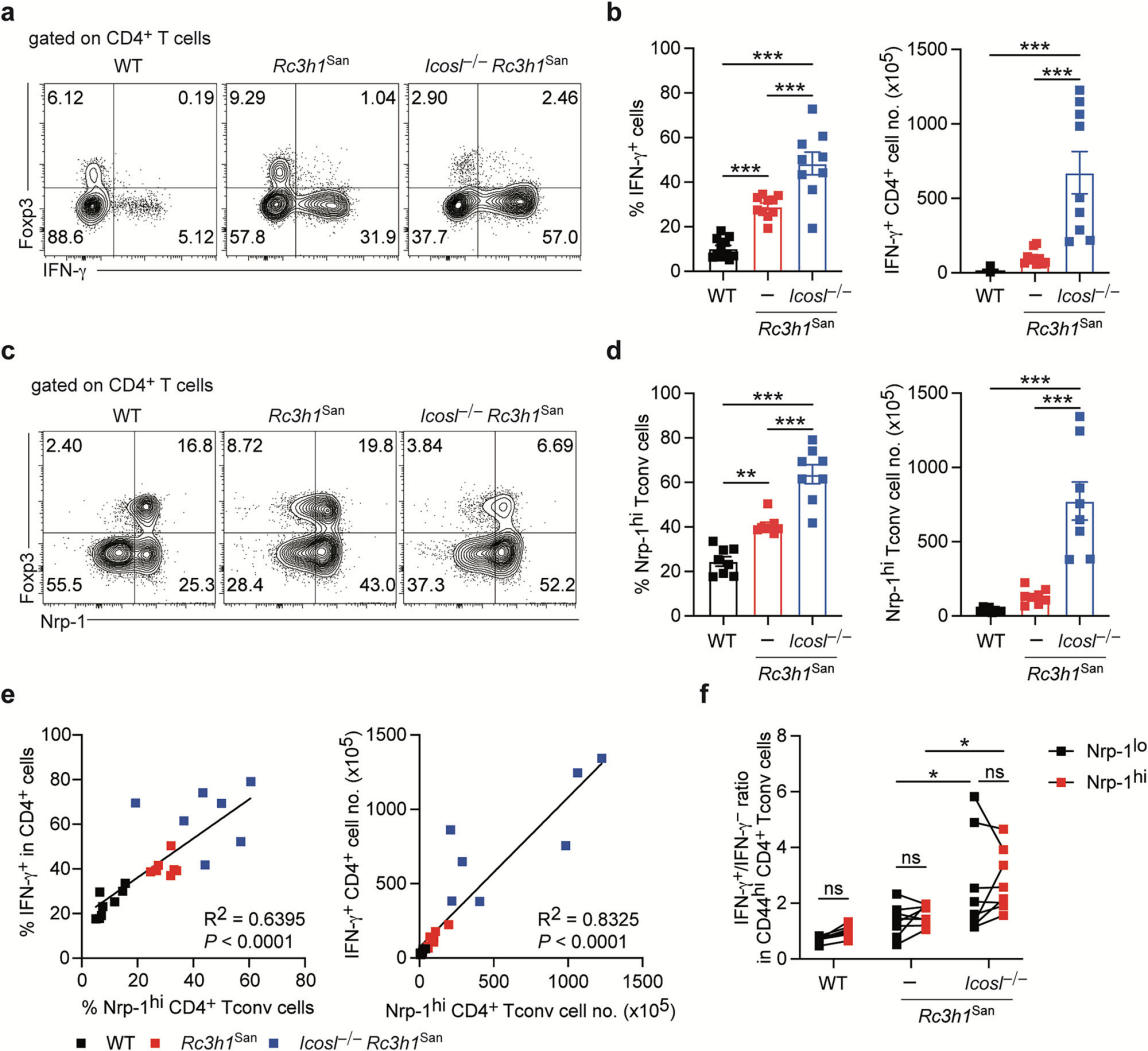
IFN-γ-producing self-reactive Nrp-1^hi^ CD4^+^ conventional T cells accumulated in *Sanroque* and inducible T cell costimulator ligand-deficient *Sanroque* mice. **a–d** Splenocytes were prepared from 16-week-old wild type (WT), *Sanroque* and inducible T cell costimulator ligand (ICOSL)-deficient *Sanroque* mice (*n* = 11 for WT, *n* = 10 for *Sanroque* and *n* = 9 for ICOSL-deficient *Sanroque* mice). **a** Representative contour plot of Foxp3 and IFN-γ gated on CD4^+^ T cells. **b** Frequency of IFN-γ^+^ cells in CD4^+^ T cells (left) and total number of IFN-γ^+^ CD4^+^ T cells (right) in the spleen. **c** Representative contour plot of Foxp3 and Nrp-1 gated on CD4^+^ T cells. **d** Frequency of Nrp-1^hi^ Foxp3^−^ cells in CD4^+^ T cells (left) and the total number of these cells in the spleen. **e** Positive correlation between the levels of IFN-γ^+^ CD4^+^ T cells and Nrp-1^hi^ Foxp3^−^ cells based on the frequencies of each population in CD4^+^ T cells (left) or the total number of each population (right) in the spleen (*n* = 8 per WT and *Sanroque*, and *n* = 7 per ICOSL-deficient *Sanroque* mice). **f** Ratio of IFN-γ^+^ and IFN-γ^−^ CD4^+^ Tconv cells within CD44^hi^ effector/memory phenotype cells based on Nrp-1 expression in the indicated groups (*n* = 8 per group). Data are pooled from two or three independent experiments. Statistical differences were determined by one-way analysis of variance with Tukey’s multiple comparisons test (**b**, **d**) or two-way analysis of variance with Bonferroni multiple comparisons test (**f**). *P < 0.05, **P < 0.01, ***P < 0.001. ns, not significant. Error bars represent SEM. Each symbol represents an individual mouse.

IFN-γ is one of the key cytokines involved in the induction of ABCs^42,43^. Moreover, augmented IFN-γ-producing Th1 cells have been shown to contribute critically to the development of SLE-like symptoms in *Sanroque* mice. Blockade of IFN-γ signalling ameliorates SLE-like disease in *Sanroque* mice^6^. Consistently, we observed that Th1 cells, already elevated in *Sanroque* mice compared with WT mice, were further increased in ICOSL-deficient *Sanroque* mice (Fig. 5a, b).

Previously, it has been proposed that Nrp-1^hi^ CD4^+^ Tconv cells increase in a SLE disease model and human SLE patients, representing self-reactive effector T cells^27^. Nrp-1^hi^ CD4^+^ Tconv cells were found even under steady-state conditions in WT mice (Fig. 5c, d). To further examine whether self-antigens drive the induction of these cells, we analysed their presence in WT mice housed under specific pathogen-free (SPF), germ-free (GF) and antigen-free (AF) conditions. AF mice were maintained under GF conditions devoid of dietary antigens by feeding elemental amino acid diet^30^. We found that the levels of Nrp-1^hi^ CD4^+^ Tconv cells were comparable among SPF, GF and AF mice (Supplementary Fig. 6a, b). The majority of Nrp-1^hi^ CD4^+^ Tconv cells were CD44^hi^ effector/memory phenotype CD4^+^ T cells, regardless of the exposure to exogenous antigens (Supplementary Fig. 6c, d). In accordance with the previous finding^27^, these results strongly support Nrp-1^hi^ CD4^+^ Tconv cells representing self-reactive CD4^+^ T cells.

The frequency of Nrp-1^hi^ CD4^+^ Tconv cells was elevated in *Sanroque* mice compared with WT mice (Fig. 5c, d). Interestingly, compared with *Sanroque* mice, the frequency and total numbers of Nrp-1^hi^ CD4^+^ Tconv cells were markedly increased in ICOSL-deficient *Sanroque* mice (Fig. 5c, d). Both the frequency and total number of these cells in the spleen were correlated well with those of IFN-γ-producing CD4^+^ T cells (Fig. 5e). Within CD44^hi^ effector/memory phenotype CD4^+^ T cells, the proportion of IFN-γ^+^ cells was comparable between Nrp-1^hi^ and Nrp-1^lo^ CD4^+^ Tconv cells, suggesting that the augmentation of IFN-γ-producing CD4^+^ T cells primarily reflects expansion of Nrp-1^hi^ CD4^+^ Tconv cells rather than enhanced IFN-γ production capacity per cell (Fig. 5f). Collectively, these findings imply a significant connection between self-reactive Nrp-1^hi^ CD4^+^ Tconv cells and the pathogenesis of SLE.

### Nrp-1^hi^ CD4^+^ conventional T cells effectively compete with Treg cells for dendritic cell engagement

Next, we investigated the underlying mechanisms responsible for the accumulation of Nrp-1^hi^ CD4^+^ Tconv cells in both *Sanroque* and ICOSL-deficient *Sanroque* mice. In mixed BM chimeras with WT and *Sanroque* BM cells, Nrp-1^hi^ CD4^+^ Tconv cells were preferentially increased in CD4^+^ T cells originating from *Sanroque* BM cells, suggesting that T cell-intrinsic factors promote the expansion of this population (Supplementary Fig. 2 f, g). Furthermore, as Nrp-1^hi^ CD4^+^ Tconv cells were not increased but rather decreased in *Icosl*^−/−^ mice, defective ICOS signalling in CD4^+^ Tconv cells was not directly responsible for the augmentation of Nrp-1^hi^ CD4^+^ Tconv cells (Supplementary Fig. 7a–c).

**Fig. 6:**
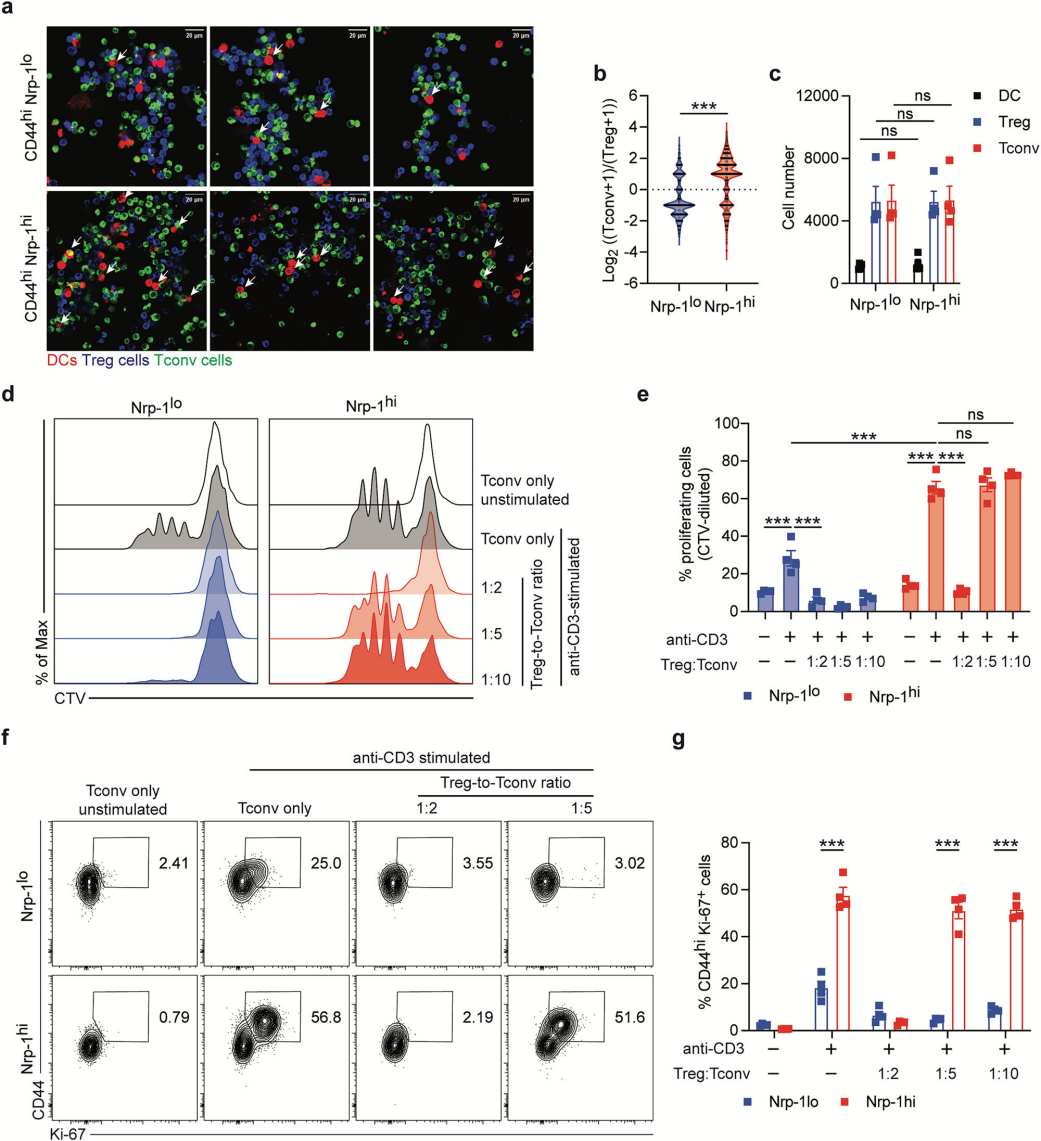
Nrp-1^hi^ CD4^+^ conventional T cells effectively compete with Treg cells for dendritic cell engagement. Splenocytes and lymph-node cells were isolated from CD45.1 *Foxp3*^GFP^ mice. Dendritic cells (DCs) (MHC-II^hi^ CD11c⁺), Treg cells (CD4⁺ TCR-β^+^ GFP⁺), and Nrp-1^lo^ and Nrp-1^hi^ CD44 ^hi^ Tconv (CD4⁺ TCR-β^+^ GFP^–^) cells were FACS sorted. **a–c** DCs, Treg cells, either Nrp-1^lo^ or Nrp-1^hi^ CD44 ^hi^ Tconv cells were labelled with lipophilic, fluorescent tracers, DiO, DiI and DiD, respectively. Lin^−^ CD11c^+^ DCs were co-cultured with Treg cells and either Nrp-1^lo^ or Nrp-1^hi^ CD44^hi^ CD4^+^ Tconv cells. Cells were cultured for 24 h. For antigenic stimulation, soluble anti-CD3ε mAbs were added to the co-culture to be displayed on DCs. **a** Representative images showing interactions between DCs and either Nrp-1^lo^ (upper panel) or Nrp-1^hi^ CD44^hi^ CD4^+^ Tconv cells (lower panel) in the presence of Treg cells. Fluorescent signals were pseudocoloured for visualization as follows: DCs, red; Treg cells, light blue; either Nrp-1^lo^ or Nrp-1^hi^ CD44^hi^ Tconv cells, green. Arrows indicate DCs that preferentially interact with either Nrp-1^lo^ or Nrp-1^hi^ CD4^+^ Tconv cells. Scale bar, 20 μm. **b** Violin plot showing the ratio of Nrp-1^lo^ and Nrp-1^hi^ CD44^hi^ Tconv cells to Treg cells among cells in close proximity to DCs. **c** Numbers of each T cell subset at the imaging time point. **d–g** DCs were co-cultured with mixtures of Treg cells and either WT Nrp-1^lo^ or Nrp-1^hi^ CD4^+^ Tconv cells at varying Treg-to-Tconv cell ratios in the presence of soluble anti-CD3ε mAbs. Nrp-1^lo^ or Nrp-1^hi^ CD4^+^ Tconv cells were labelled with CTV dye to measure cell division. Cells were cultured for 3 days. **d** Representative histograms showing CTV dilution of WT Nrp-1^lo^ (left) or Nrp-1^hi^ CD4^+^ Tconv cells (right) under indicated conditions. **e** Frequency of CTV-diluted proliferating CD4^+^ Tconv cells. **f** Representative contour plots showing CD44 and Ki67 expression in Nrp-1^lo^ or Nrp-1^hi^ CD4^+^ Tconv cells. **g** Frequency of CD44^hi^ Ki67^+^ cells. Two independent experiments show similar results. Statistical differences were determined by unpaired two-tailed Student’s *t* test (**b**) or two-way analysis of variance with Bonferroni multiple comparisons test (**c**, **e**, **g**). **P < 0.01, ***P < 0.001. ns, not significant. Error bars represent SEM. Each symbol represents a biological replicate.

Previous studies have shown that Treg cells inhibit the interaction between dendritic cells (DCs) and naïve CD4^+^ Tconv cells, and that Nrp-1, which is highly expressed on thymic Treg cells^30^, facilitated prolonged interactions with DCs^44,45^. To elucidate whether Nrp-1^hi^ CD4^+^ Tconv cells can compete with Treg cells for DC engagement, we conducted an in vitro interaction assay using live confocal imaging. Interestingly, in the presence of Treg cells, Nrp-1^hi^ CD4^+^ Tconv cells exhibited a stronger capacity to interact with DCs than Nrp-1^lo^ counterparts (Fig. 6a, b). Quantitative tile scan analysis further revealed that individual DCs interacted more frequently with Nrp-1^hi^ CD4^+^ Tconv cells than with Treg cells (Fig. 6a, b). These findings were not biased by the alteration in subset abundance and viability, as the numbers of each T cell subset and the relative ratios of Treg cells to either Nrp-1^lo^ or Nrp-1^hi^ CD4^+^ Tconv cells were comparable at the imaging time point (Fig. 6c).

Next, we examined whether the enhanced capacity of Nrp-1^hi^ CD4^+^ Tconv cells for DC engagement over Treg cells results in enhanced proliferation of Nrp-1^hi^ CD4^+^ Tconv cells, even in the presence of Treg cells. Using a CellTrace Violet (CTV) dilution assay, we measured the proliferative capacity of Nrp-1^lo^ or Nrp-1^hi^ CD4^+^ Tconv cells co-cultured with Treg cells at varying Treg-to-Tconv cell ratios and DCs treated with soluble anti-CD3ε mAb. In the absence of Treg cells, Nrp-1^hi^ CD4^+^ Tconv cells proliferated more effectively than Nrp-1^lo^ counterparts (Fig. 6d, e). Notably, although Treg cells effectively suppressed CD4^+^ Tconv cell proliferation at a higher Treg-to-Tconv cells ratio (1:2), Nrp-1^hi^ CD4^+^ Tconv cells preferentially escaped from Treg-mediated suppression at lower ratios of Treg-to-Tconv cells (Fig. 6d, e). The frequency of CD44^hi^ Ki67^+^ cells also correlated well with the proliferative capacity of Nrp-1^hi^ CD4^+^ Tconv cells (Fig. 6f, g). Collectively, these findings suggest that Nrp-1^hi^ CD4^+^ Tconv cells retain a competitive proliferative advantage under Treg cell-mediated suppression, at least in part because of their enhanced capacity for DC engagement.

### Nrp-1^hi^ CD4^+^ conventional T cells exhibit enhanced dendritic cell engagement despite sharing antigen specificity with Treg cells

Considering that both Treg cells and Nrp-1^hi^ CD4^+^ Tconv cells are enriched for self-antigen recognition, the enhanced DC engagement capacity of Nrp-1^hi^ CD4^+^ Tconv cells over Nrp-1^lo^ CD4^+^ Tconv cells could potentially reflect differences in TCR specificity between these subsets. To compare DC engagement by Treg cells and either Nrp-1^lo^ or Nrp-1^hi^ CD4^+^ Tconv cells in an antigen-matched setting, we analysed DC–T cell engagement using CD4^+^ T cells from B6 OT-II TCR-transgenic (Tg) mice. CD4^+^ T cells from these TCR-Tg mice specifically recognize ovalbumin (OVA)_323-339_ peptide presented on MHC-II molecules. Compared with OT-II TCR-Tg mice against a *Rag1*^−/−^ background, B6 OT-II Tg mice have CD44^hi^ effector/memory phenotype CD4^+^ T cells, presumably because of the dual TCR expression^46^. Therefore, we were able to obtain CD44^hi^ Nrp-1^lo^ or CD44^hi^ Nrp-1^hi^ OT-II cells.

Consistent with our earlier findings, CD44^hi^ Nrp-1^hi^ OT-II cells interacted more efficiently with OVA_323__–339_ peptide-loaded DCs than their Nrp-1^lo^ counterparts in the presence of OT-II Treg cells (Supplementary Fig. 8a, b). In the absence of OVA_323__–339_ peptide pulsing, T cells that engaged with DCs were markedly reduced. However, even after 12 h of co-culture, DC–T cell engagement was significantly increased upon treatment with OVA_323__–339_ peptides, indicating that OT-II Tg TCR recognition of the OVA_323-339_ peptide–MHC-II (pMHC-II) complex on DCs is the dominant driver of DC–T cell engagement rather than endogenous TCR–pMHC-II interactions (Supplementary Fig. 8d, e). Importantly, even in these antigen-matched conditions, Nrp-1^hi^ OT-II cells still engaged DCs more efficiently than Nrp-1^lo^ counterparts (Supplementary Fig. 8d, e). These results were also not attributable to the differences in subset abundance and viability (Supplementary Fig. 8c, f). Collectively, these findings suggest that DC–T engagement is dependent on TCR–pMHC-II interaction, and that the competitive advantage of Nrp-1^hi^ CD4^+^ Tconv cells in DC engagement over Treg cells is not caused by intrinsic differences in TCR specificity.

### Nrp-1^hi^ CD4^+^ conventional T cells promote the induction of age-associated B cells

Previously, it was reported that PD-1^hi^ CXCR5^−^ CD4^+^ T cells, known as Tph cells, drive B cell activation in GC-independent manner, including the induction of ABCs^26,47^. Given these findings, we examined whether IFN-γ-producing Nrp-1^hi^ CD4^+^ Tconv cells, which were augmented in ICOSL-deficient *Sanroque* mice, share functional traits with Tph cells.

Even under steady-state conditions in B6 mice, Nrp-1^hi^ CD4^+^ Tconv cells exhibited elevated PD-1 expression compared with CD44^hi^ Nrp-1^lo^ counterparts, with most Nrp-1^hi^ CD4^+^ Tconv cells lacking CXCR5 expression. In *Sanroque* mice, both PD-1^hi^ CXCR5^+^ Tfh cells and PD-1^hi^ CXCR5^−^ Tph cells were more prominently enriched within Nrp-1^hi^ CD4^+^ Tconv cells compared with the Nrp-1^lo^ counterparts (Supplementary Fig. 9a, b). As expected, Nrp-1^hi^ CD4^+^ Tconv cells in ICOSL-deficient *Sanroque mice* expressed PD-1 at a higher level than Nrp-1^lo^ counterparts and also lacked CXCR5 expression, supporting their potential role as self-reactive Tph cells (Supplementary Fig. 9a, b).

**Fig. 7:**
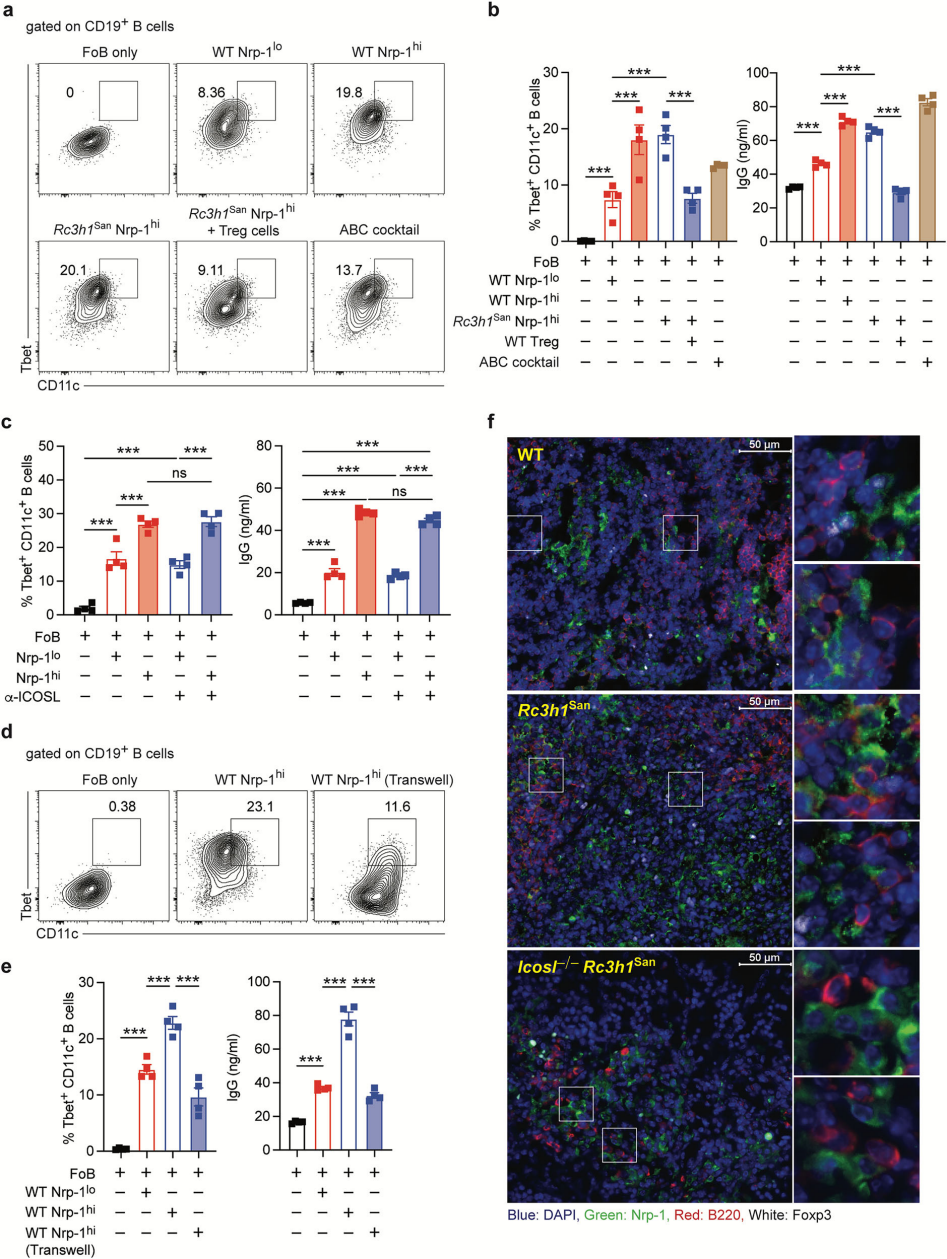
Nrp-1^hi^ CD4^+^ conventional T cells promote the differentiation of age-associated B cells. CD23^+^ CD21^int^ follicular B (FoB) cells were FACS-sorted from the spleen of 6–7-week-old wild type (WT) B6 mice. As a negative control, FoB cells were stimulated with agonistic anti-IgM and anti-CD40 mAbs. For co-culture experiments, FoB cells were cultured with either Nrp-1^lo^ or Nrp-1^hi^ CD44^hi^ CD4^+^ Tconv cells from WT B6 or *Sanroque* mice. T cells were activated using plate-bound anti-CD3ε and anti-CD28 mAbs. To examine Treg-mediated suppression of age-associated B cell (ABC) differentiation, WT Treg cells were added. As a positive control for ABC differentiation, FoB cells were stimulated with anti-mouse IgM, and anti-CD40 antibodies in the presence of an ABC-inducing cocktail (ABC cocktail) containing imiquimod, recombinant IL-21 and IFN-γ. **a** Representative contour plot showing Tbet^+^ CD11c^+^ ABCs gated on CD19^+^ B cells. **b** Frequency of ABCs (left) and IgG levels in culture supernatants (right) in the indicated groups. **c** Anti-ICOSL antagonistic mAb was added to examine the role of ICOSL signalling in ABC differentiation by Nrp-1^hi^ CD44^hi^ CD4^+^ Tconv cells from WT mice. Frequency of ABCs (left) and IgG levels in culture supernatants (right) in the indicated groups. **d**, **e** Transwell experiment to examine contact-dependent enhancement of ABC induction by Nrp-1^hi^ CD4^+^ Tconv cells. WT Nrp-1^hi^ CD44^hi^ CD4^+^ Tconv cells were seeded in the lower chamber pre-coated with anti-CD3ε and anti-CD28 mAbs, and CD23⁺ CD21^int^ FoB cells were in the upper transwell insert. Cells were cultured for 3 days in complete RPMI-1640 medium and harvested for analysis. **d** Representative contour plot showing Tbet^+^ CD11c^+^ ABCs gated on CD19^+^ B cells. **e** Frequency of ABCs (left) and IgG levels in culture supernatants (right) in the indicated groups. **f** Representative immunofluorescence image of spleen sections from the indicated mice showing Treg cells, B cells and Nrp-1^hi^ cells. The scale bar indicates 50 μm (left). The boxed regions were shown as magnified views (right). DAPI is shown in blue, B220 in red, Nrp-1 in green and Foxp3 in white. At least two independent experiments show similar results. Statistical differences were determined by one-way analysis of variance with Tukey’s multiple comparisons test (**b**, **c**, **e**). *P < 0.05, **P < 0.01, ***P < 0.001. Error bars represent SEM. Each symbol represents a biological replicate.

To determine whether Nrp-1^hi^ CD4^+^ Tconv cells can function similarly to Tph cells and promote ABC induction, we co-cultured either CD44^hi^ Nrp-1^lo^ or CD44^hi^ Nrp-1^hi^ CD4^+^ Tconv cells with CD11b^−^ CD11c^−^ CD19^+^ ~~CD23^+^ CD21^int^ follicular B cells (FoBs) in vitro in the presence of anti-CD3ε and anti-CD28 agonistic mAbs. We found that Nrp-1^hi^ CD4^+^ Tconv cells more effectively induced CD11c^+^ Tbet^+^ ABCs than their Nrp-1^lo^ counterparts (Fig. 7a, b). As a result, Nrp-1^hi^ CD4^+^ Tconv cells significantly enhanced IgG production by ABCs compared with Nrp-1^lo^ counterparts (Fig. 7b). Nrp-1^hi^ CD4^+^ Tconv cells in *Sanroque* mice also promoted ABC differentiation and IgG production as effectively as WT Nrp-1^hi^ CD4^+^ Tconv cells (Fig. 7a, b). Interestingly, Treg cells effectively inhibit ABC differentiation induced by Nrp-1^hi^ CD4^+^ Tconv cells and subsequently suppressed IgG production (Fig. 7a, b). In accordance with the augmentation of ABCs in ICOSL-deficient *Sanroque* mice, blocking ICOS signalling did not impair the ability of Nrp-1^hi^ CD4^+^ Tconv cells to induce ABCs, suggesting that ICOS/ICOSL interaction between CD4^+^ T cells and B cells is not essential for ABC induction (Fig. 7c).

Nrp-1^hi^ CD4^+^ Tconv cells effectively produce IFN-γ, which promotes ABC induction^42,43^. However, IFN-γ production is not solely attributable to enhanced ABC induction by Nrp-1^hi^ CD4^+^ Tconv cells, as IFN-γ production was comparable between Nrp-1^lo^ and Nrp-1^hi^ CD4^+^ Tconv cells (Fig. 5f). Indeed, the in vitro transwell experiment showed that spatially separated Nrp-1^hi^ CD4^+^ Tconv cells failed to enhance ABC induction, suggesting that Nrp-1^hi^ CD4^+^ Tconv cells promote ABC induction in a contact-dependent manner (Fig. 7d, e). Furthermore, although Foxp3^−^ Nrp-1^hi^ cells were found in both B cell follicles and T cell zones in the spleen of *Sanroque* mice, Foxp3^−^ Nrp-1^hi^ cells in ICOSL-deficient *Sanroque* mice were strongly co-localized with B cells (Fig. 7f). Foxp3^−^ Nrp-1^hi^ cells in contact with B cells in ICOSL-deficient *Sanroque* mice were predominantly CD4^+^ T cells (Supplementary Fig. 10). Collectively, these results suggest that Nrp-1^hi^ CD4^+^ Tconv cells can act as Tph cells to promote the induction of ABCs in a contact-dependent manner.

## Discussion

In this study, we demonstrated that Treg cells in *Sanroque* mice expand during the onset of SLE-like disease in an ICOS signalling-dependent manner and retain intact suppressive activity. Our findings based on *Sanroque* and ICOSL-deficient *Sanroque* mice propose that self-reactive Nrp-1^hi^ CD4^+^ Tconv cells are resistant to Treg-mediated suppression of DC engagement, thereby promoting their augmentation in *Sanroque* mice. In addition, defective ICOS signalling further augments the Nrp-1^hi^ CD4^+^ Tconv cells by impairing Treg cell maintenance, without lowering aberrant antibody responses due to the induction of ABCs.

SLE is typically associated with aberrant Tfh cell-driven GC responses, which subsequently mediate the production of autoreactive IgG antibodies. In SLE, autoantibodies against nuclear components, such as DNA and histone, correlate with disease severity, and play a key role in tissue damage through the formation of immune complexes^48^. Therapeutic effects of belimumab, a monoclonal antibody targeting B cell activating factor (BAFF), in many SLE patients also underscore the importance of B cells in SLE pathogenesis^49,50^. Accordingly, targeting Tfh cells and disrupting T and B cell interaction hold promise for SLE treatment. Considering that ICOS signalling is critical for the induction of Tfh cells and the generation of high-affinity autoreactive antibodies^13,14^, blocking ICOS/ICOSL interactions can be beneficial for treating SLE. However, our findings reveal a potential caveat: targeting ICOS/ICOSL interaction in SLE might have dual effects, especially in SLE subtypes associated with augmented Treg cell population, as it could lead to a substantial reduction in Treg cells and subsequent activation of non-GC B cell responses. Indeed, targeting ICOS signalling with anti-ICOSL mAb (prezalumab, formerly AMG557) treatment in SLE patients tended to acquire clinical benefit, but improvement of disease scores did not reach statistical significance^51^.

Our study reveals that autoreactive IgG, including dsDNA-specific IgG, can be effectively generated even in the absence of Tfh cells and GC responses, as observed in ICOSL-deficient *Sanroque* mice. It is conceivable that non-GC response dominates in ICOSL-deficient *Sanroque* mice, contrasting with GC response dominance in *Sanroque* mice (Supplementary Fig. 11). Interestingly, both GC- and non-GC-driven responses appear to induce comparable levels of autoantibodies and pathological symptoms in multiple organs. Previous studies have proposed that non-GC responses compensate for the absence of Tfh cell-mediated help for the production of autoreactive antibodies in SLE-like disease models^52–54^. Moreover, inhibiting Tfh cell and GC responses redirects pathogenic responses through the non-GC responses. Our findings are consistent with these observations but offer a new perspective by demonstrating that ABCs can be generated and drive autoreactive IgG production independently of Tfh cell and GC responses. This contrasts with the requirement of Tfh cells outside of GCs to induce ABCs in a viral infection^55^. Our findings also emphasize that targeting strategies that not only block GC responses but also block non-GC responses are important for the effective treatment of SLE.

Defective ICOS signalling in *Sanroque* mice resulted in the augmentation of Th1 cells, concurrently associated with heightened expression of Nrp-1. Nrp-1^hi^ CD4^+^ Tconv cells are detectable even under homeostatic conditions, displaying a CD44^hi^ effector/memory phenotype. Notably, the emergence of Nrp-1^hi^ CD4^+^ Tconv cells does not hinge upon exogenous antigen stimulation, as evidenced by their presence in mice raised under conditions devoid of foreign antigens derived from commensal microbiota and food antigens. These observations corroborate previous findings suggesting that Nrp-1^hi^ CD4^+^ Tconv cells represent self-reactive CD4^+^ T cells^27^.

In *Sanroque* mice, the expansion of Nrp-1^hi^ CD4^+^ Tconv cells primarily occurs through a T cell-intrinsic mechanism and is associated with enhanced DC engagement compared with their Nrp-1^lo^ counterparts. T cell-intrinsic and ICOS signalling-independent mechanisms driving the accumulation of self-reactive Nrp-1^hi^ CD4^+^ Tconv cells in *Sanroque* mice remain incompletely understood. However, our findings suggest that competition with Treg cells for DC engagement is a key contributing factor. Specifically, the elevated Nrp-1 expression on self-reactive effector CD4^+^ T cells facilitates more stable interactions with antigen-presenting DCs, even in the presence of Nrp-1-expressing Treg cells. In line with previous findings^44^, our observations propose that Nrp-1 not only serves as a marker for self-reactivity but also functionally promotes pathogenic CD4^+^ T cell responses through augmented DC engagement. Nrp-1^hi^ CD4^+^ Tconv cells do not appear to be fully resistant to Treg cell-mediated suppression, presumably because of multiple suppressive mechanisms of Treg cells. This may explain the further escalation in Nrp-1^hi^ CD4^+^ Tconv cells observed in ICOSL-deficient *Sanroque* mice, in which Treg cell abundance is reduced.

The augmentation of IFN-γ-producing Nrp-1^hi^ CD4^+^ Tconv cells potentially contributes to autoreactive antibody production. Tissue damage resulting from T cell-mediated inflammation can release endogenous Toll-like receptor (TLR) ligands, such as nucleic acids, which may in turn stimulate B cells independently of T cell help. Although this mechanism cannot be entirely excluded, our findings suggest that Nrp-1^hi^ CD4^+^ Tconv cells can act as PD-1^hi^ CXCR5^−^ Tph cells, which effectively drive the induction of ABCs and facilitate autoreactive IgG production in the absence of Treg cells. Although the levels of PD-1^hi^ CXCR5^−^ cells among Nrp-1^hi^ CD4^+^ Tconv cells were comparable between *Sanroque* mice and ICOSL-deficient *Sanroque* mice, the proportion of ABCs among B cell populations was significantly increased in ICOSL-deficient *Sanroque* mice. According to our findings, this discrepancy can be explained, as ABC induction, mediated by Nrp-1^hi^ CD4^+^ Tconv cells, was effectively suppressed by Treg cells. Interestingly, the proportion of PD-1^hi^ CXCR5^+^ Tfh cells is higher among Nrp-1^hi^ CD4^+^ Tconv cells than among Nrp-1^lo^ counterparts in *Sanroque* mice, presumably because of the enhanced capacity of Nrp-1^hi^ CD4^+^ Tconv cells for DC engagement. Given that Nrp-1 is not expressed in human Treg cells and Nrp-1^hi^ CD4^+^ Tconv cells are increased in patients with SLE^27^, Nrp-1^hi^ CD4^+^ Tconv cells can be a therapeutic target for simultaneously suppressing both GC and non-GC responses in SLE.

Previous studies have shown that Nrp-1 can function as a stabilizer that promotes cell–cell adhesion. Nrp-1 acts as a coreceptor for multiple cell surface receptors and can interact with adhesion molecules, including integrins^56^. Notably, Nrp-1 has been reported to stabilize the immunological synapse in conjunction with LFA-1 (ref. ^44^), which is critical for the interaction of T cells with both DCs and B cells^44,57^. Although we did not define the precise molecular components mediating the interactions between Nrp-1^hi^ CD4^+^ Tconv and DCs or B cells, our findings that Nrp-1^hi^ CD4^+^ Tconv cells promote ABC induction in vitro in a contact-dependent manner, together with their preferential engagement with B cells in vivo in ICOSL-deficient *Sanroque* mice, are consistent with previous reports. These observations support the idea that Nrp-1-dependent enhancement of cell–cell adhesion, either directly or indirectly, represents a key mechanism promoting at least non-GC responses in SLE.

We observed that *Sanroque* Treg cells are more liable to become ex-Treg cells under lymphopenic conditions. Given the high affinity of Treg cells for self-antigens, the conversion of Treg cells into effector CD4^+^ T cells could potentially exacerbate the autoimmune disease pathogenesis. Currently, it remains unclear whether ex-Treg cells may contribute to the augmentation of self-reactive Nrp-1^hi^ CD4^+^ Tconv cells in *Sanroque* mice. Intriguingly, both populations share common attributes, including specificity to self-antigens and reliance on the nuclear receptor 4A2 (NR4A2) transcription factor. NR4A2 is known to be essential not only for the induction of Nrp-1^hi^ CD4^+^ Tconv cells^27^ but also for the induction and functions of Treg cells^58^. Further investigations employing the fate-mapping analyses or comparative analyses of TCR repertoires between the two populations are required to determine whether Treg-to-effector conversion directly contributes to SLE pathogenesis.

In conclusion, our study provides new insights into the dual role of ICOS signalling in regulating SLE by maintaining Treg cell populations and orchestrating GC and non-GC B cell responses. Although ICOS blockade shows therapeutic promise by suppressing GC responses, its impact on Treg cells and subsequent influences on non-GC responses warrants caution. These findings emphasize the importance of comprehensive strategies targeting multiple immune pathways to block autoantibody production in SLE. Notably, Nrp-1^hi^ CD4^+^ Tconv cells, encompassing both Tfh and Tph cell subsets, emerge as a pivotal contributor to autoreactive antibody production and represent a promising target for therapeutic intervention in SLE.

## Supplementary information

Supplementary Information

## Data Availability

All data needed to evaluate the conclusions in the paper are present in the paper and/or the Supplementary materials. Additional data related to this paper may be requested from the authors.
