# Supplementary material for: Defective inducible T cell costimulator signalling drives neuropilin-1hi CD4+ T cell-mediated non-germinal centre B cell activation via impaired regulatory T cell maintenance in a lupus model
Source: Exp Mol Med. 2026 Jun 10;58(6):1971–86. doi: 10.1038/s12276-026-01756-6 (PMC13324385; doi:10.1038/s12276-026-01756-6)
Supplement: Supplementary file 1 — Supplementary Information [file 12276_2026_1756_MOESM1_ESM.docx]

**SUPPLEMENTARY INFORMATION**

**Defective inducible T cell costimulator signalling drives neuropilin-1^hi^ CD4^+^ T cell-mediated non-germinal centre B cell activation via impaired regulatory T cell maintenance in a lupus model**

Sumin Kang^1^, Da Eun Kim^2^, Nahyun Kim^2^, Eunju O^2^, Hye Jin Yoon^1^, Yesung Joo^3^, Sung-Wook Hong^2, 3^ and Kwang Soon Kim^1, 2,^ *

^1^Department of Life Sciences, Pohang University of Science and Technology (POSTECH), Pohang, Republic of Korea

^2^Department of Integrative Biosciences and Biotechnology, Pohang University of Science and Technology (POSTECH), Pohang, Republic of Korea

^3^Department of Biotechnology, College of Life Science and Biotechnology, Yonsei University, Seoul, Republic of Korea

*Correspondence should be addressed to

Dr. Kwang Soon Kim, Department of Life Sciences. Pohang University of Science and Technology (POSTECH), Pohang, 37673, Republic of Korea, Email: kskim27@postech.ac.kr, Dr. Sung-Wook Hong, Department of Biotechnology, Yonsei University, Seoul, 03722, Republic of Korea, Email: hongsw@yonsei.ac.kr

**SUPPLEMENTARY FIGURES**


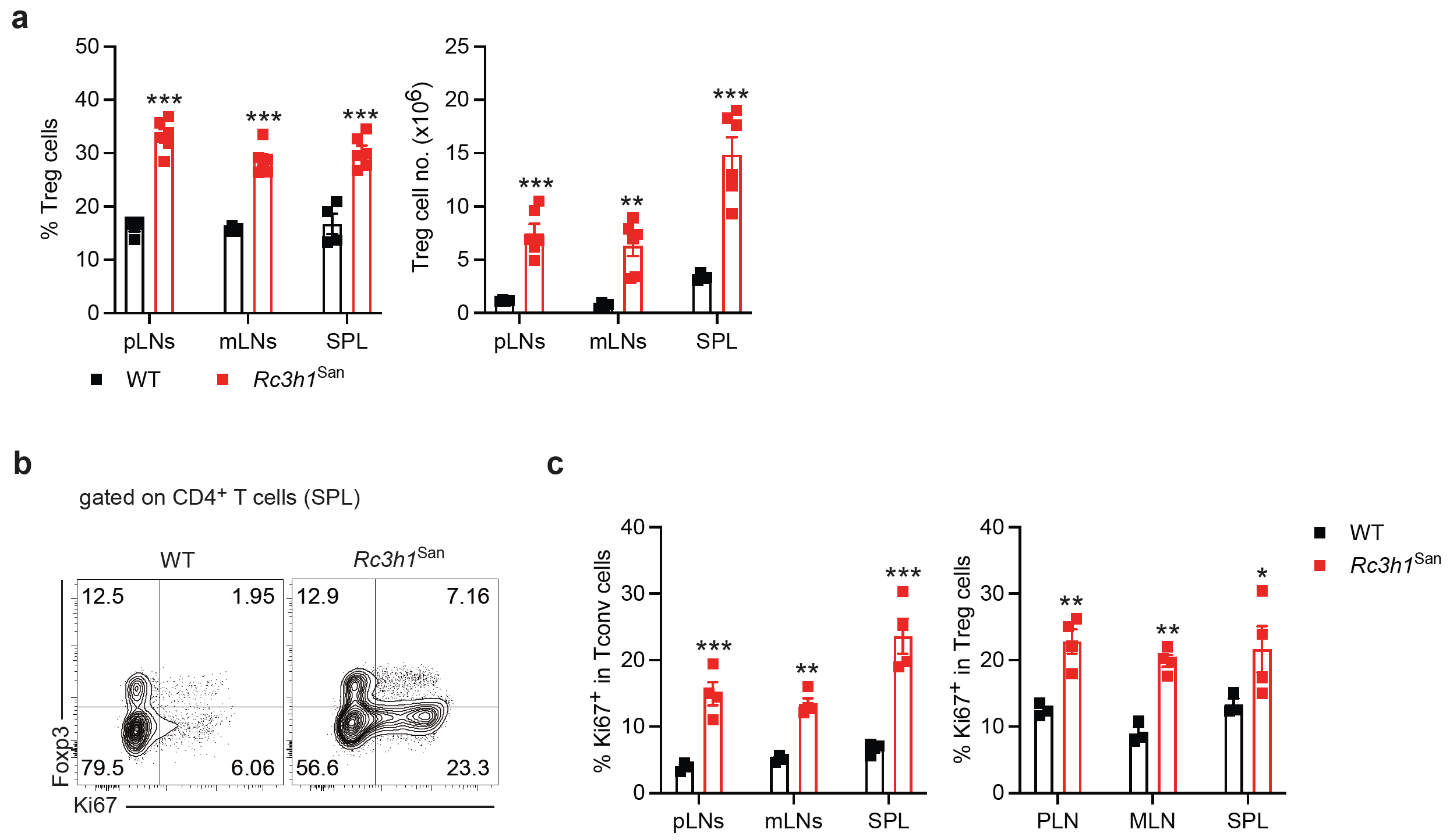


**Supplementary Fig. 1. Foxp3^+^ regulatory CD4^+^ T cells proliferate more vigorously in *Sanroque* mice.** (a) Frequency of Foxp3^+^ Treg cells among CD4^+^ T cells (left) and total number of Treg cells in the indicated tissues from 8-week-old WT and *Sanroque* (*Rc3h1*^San^) mice (n=4 for WT and n=6 for *Sanroque* mice). (b) Representative contour plots of Foxp3 and Ki67 gated on splenic CD4^+^ T cells in WT and *Sanroque* mice. (c) Frequency of Ki67^+^ cells in CD4^+^ Tconv cells (left) and in Treg cells (right) from the spleen of WT and Sanroque mice (n=3 for WT and n=4 for *Sanroque* mice). At least, two independent experiments show similar results. Statistical differences were determined by unpaired two-tailed Student’s *t* test (a, c). **p*<0.05, ***p*<0.01, ****p*<0.001. Error bars represent SEM. Each symbol represents an individual mouse.

**
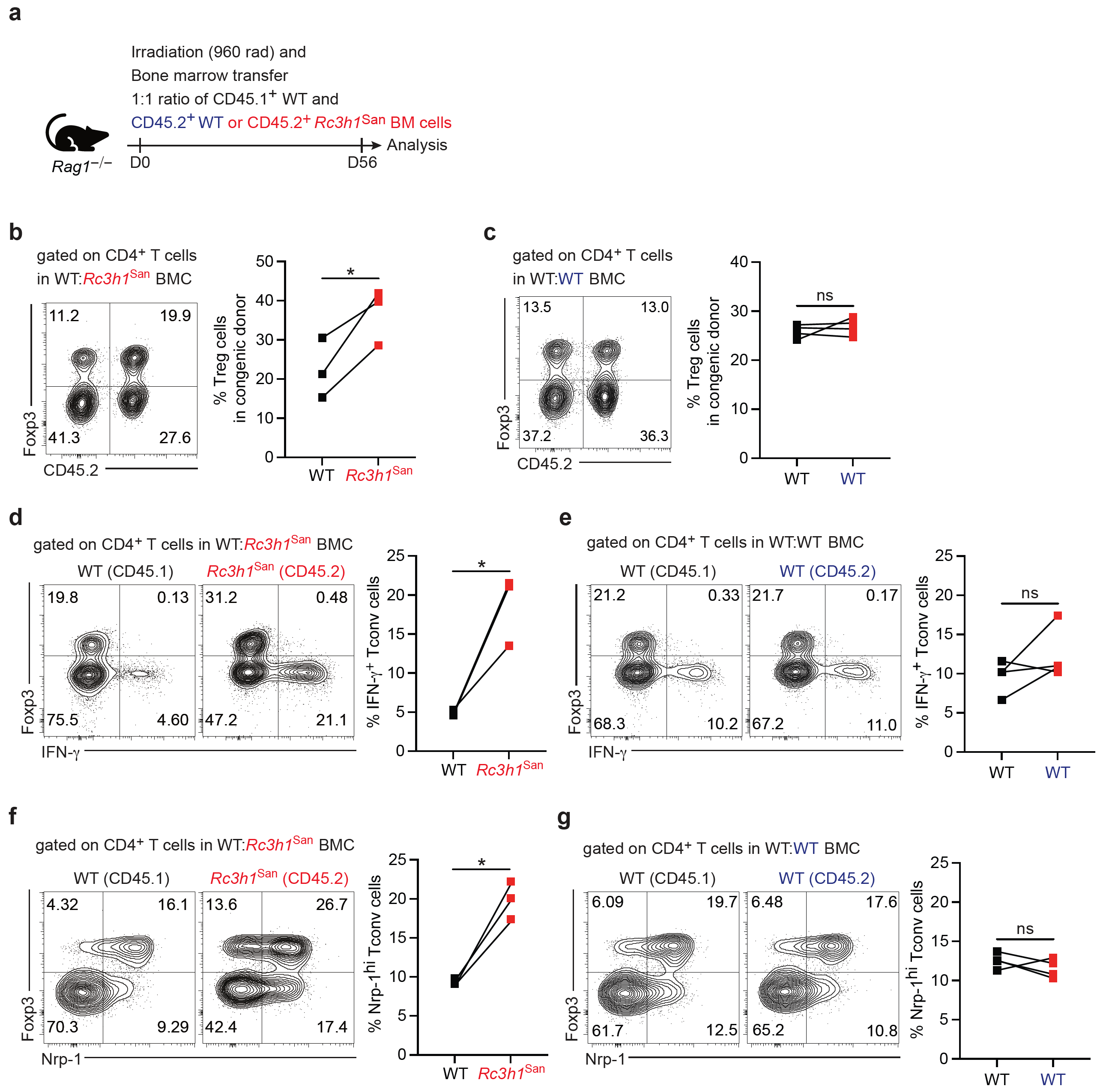
**

**Supplementary Fig. 2. T cell phenotypes in spleen from WT/WT or WT/*Sanroque* mixed bone marrow chimeras.** *Rag1*^−/−^ mice were irradiated at a dose of 6 Gy and then transplanted with a 1:1 mixture of bone marrow cells from CD45.1^+^ WT and either CD45.2^+^ WT or *Sanroque* (*Rc3h1*^San^) mice (n=3 per group). Mice were analysed 8 weeks after bone marrow reconstitution. **(**a) Experimental scheme of mixed bone marrow chimera (BMC). (b-c) Representative contour plots showing Treg cells (left) and frequency of Treg cells gated on each congenic donor CD4^+^ T cells (right) in the spleen of WT/*Sanroque* (b) and WT/WT (c) BMC. (d-e) Representative contour plots of Foxp3 and IFN-γ (left) and frequency of IFN-γ^+^ CD4^+^ Tconv cells (right) gated on each congenic donor CD4^+^ T cells in WT/*Sanroque* (d) and WT/WT (e) BMC. (f-g) Representative contour plots of Foxp3 and Nrp-1 (left) and frequency of Nrp-1^+^ CD4^+^ Tconv cells (right) gated on each congenic donor CD4^+^ T cells in WT/Sanroque (f) and WT/WT (g) BMC. Statistical differences were determined by paired two-tailed Student’s *t* test. **p*<0.05, ns: not significant. Error bars represent SEM. Each symbol represents an individual mouse.

**
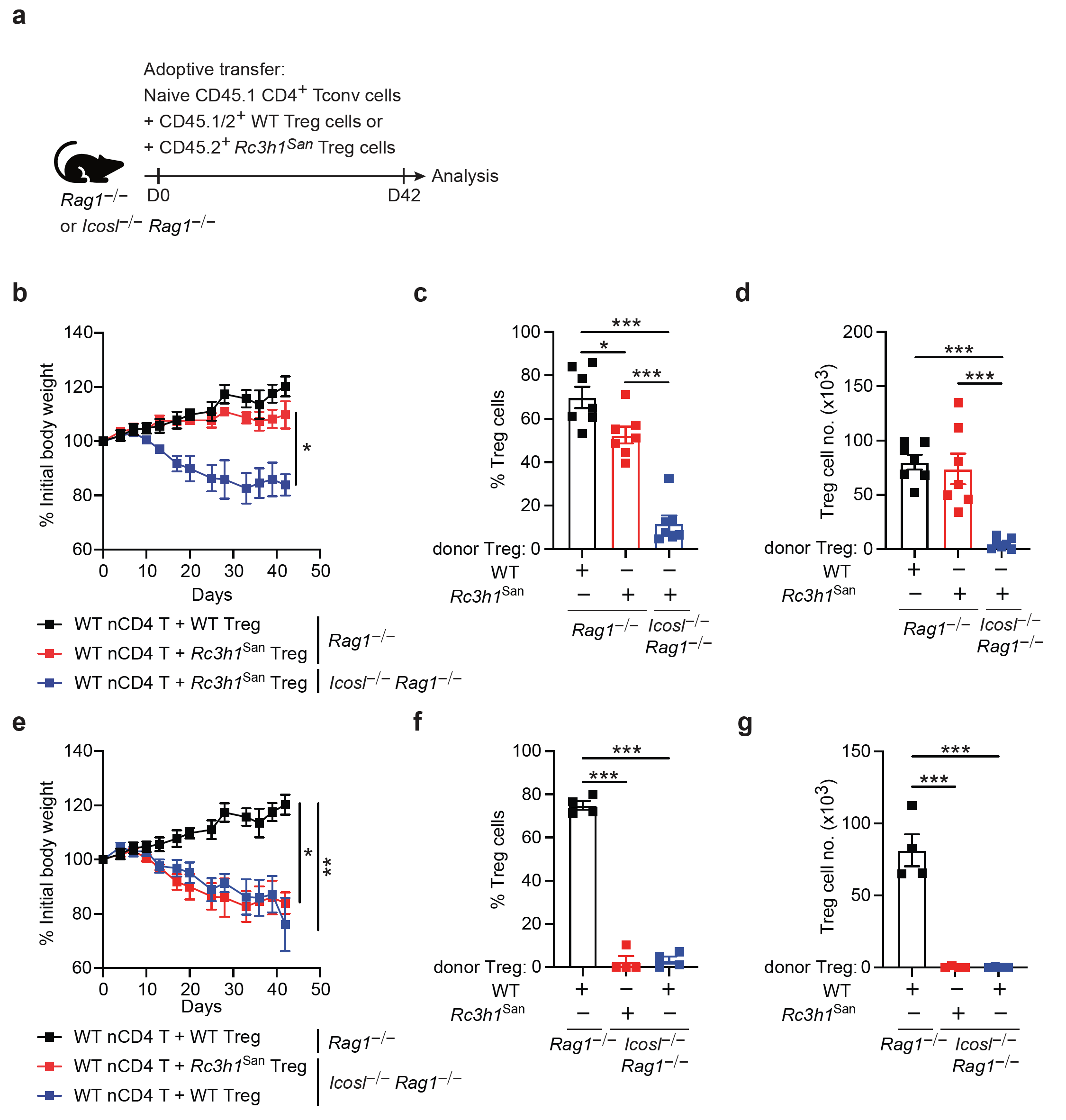
**

**Supplementary Fig. 3. Treg cells are unstable in ICOSL-deficient lymphopenic mice.** Either CD45.1/CD45.2^+^ WT Treg cells (WT Treg) or CD45.2^+^ *Sanroque* Treg (*Rc3h1*^San^ Treg) cells were co-transferred into *Rag1*^−/−^ mice or *Icosl*^−/−^ *Rag1*^−/−^ mice with CD45.1^+^ naïve WT CD4^+^ Tconv (nCD4 T) cells. (a) Experimental scheme of adoptive T cell transfer. (b) Initial body weight changes after adoptive transfer in *Rag1*^−/−^ mice transferred with WT CD4^+^ Tconv cells and either WT or *Sanroque* Treg cells and *Icosl*^−/−^ *Rag1*^−/−^ mice transferred with WT CD4^+^ Tconv cells and *Sanroque* Treg cells (n=7 per group). (c-d) Frequency of donor Treg cells in donor CD4^+^ T cells (c) and total number of donor Treg cells (d) in the spleen of indicated mice at day 42 after adoptive transfer. Data are pooled from two independent experiments. (e) Initial body weight changes after adoptive transfer in *Rag1*^−/−^ mice transferred with WT CD4^+^ Tconv cells and WT Treg cells and *Icosl*^−/−^ *Rag1*^−/−^ mice transferred with WT CD4^+^ Tconv cells and either WT or *Sanroque* Treg cells (n=4 per group). (f-g) Frequency of donor Treg cells in donor CD4^+^ T cells (f) and total number of donor Treg cells (g) in the spleen of indicated mice at day 42 after adoptive transfer. Statistical differences were determined by one-way ANOVA with Tukey's multiple comparisons test. **p*<0.05, ****p*<0.001. Error bars represent SEM. Each symbol represents an individual mouse.

**
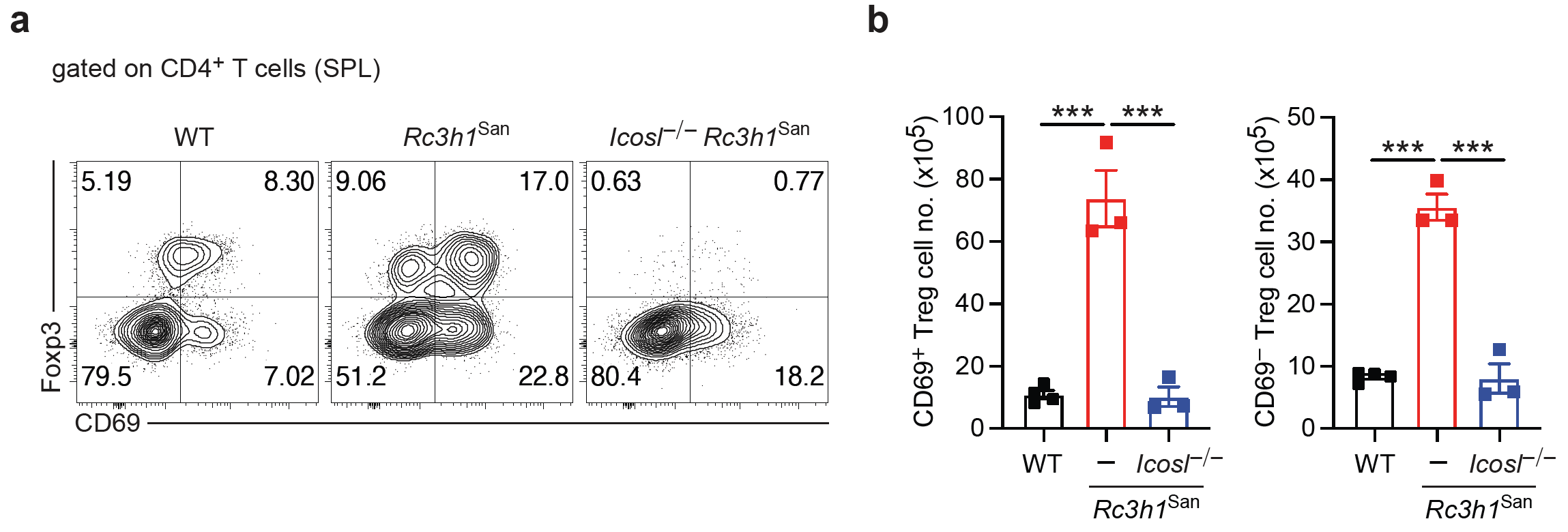
**

**Supplementary Fig. 4. Levels of CD69+ Treg cells in ICOSL-deficient Sanroque mice.** (a) Representative contour plots of Foxp3 and CD69 gated on splenic CD4^+^ T cells in 16-week-old WT, Sanroque (*Rc3h1*^San^) and ICOSL-deficient Sanroque (*Icosl*^−/−^ *Rc3h1*^San^) mice. (b) Total number of CD69^+^ (left) and CD69^−^ (right) Treg cells in the spleen of the indicated mice (n=3 per group). Two independent experiments show similar results. Statistical differences were determined by one-way ANOVA with Tukey's multiple comparisons test. ****p*<0.001. Error bars represent SEM. Each symbol represents an individual mouse.

**
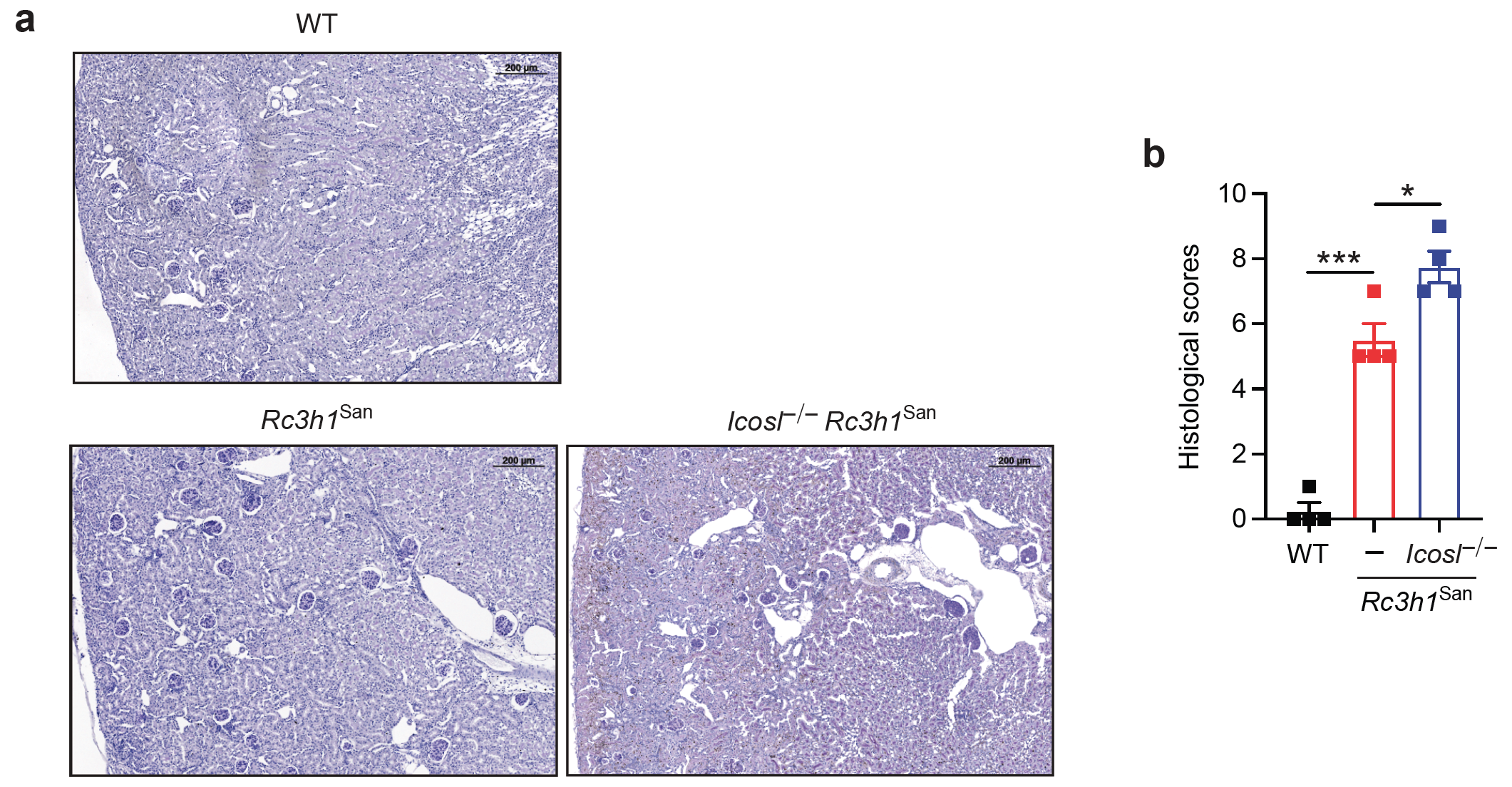
**

**Supplementary Fig. 5. Kidney inflammation in WT, *Sanroque* and ICOSL-deficient *Sanroque* mice.** (a) Representative periodic acid–Schiff (PAS)-stained kidney sections from 16-week-old WT, Sanroque (*Rc3h1*^San^) and ICOSL-deficient Sanroque (*Icosl*^−/−^ *Rc3h1*^San^) mice. Scale bar, 200 µm. (b) Histological scores of kidney sections from the indicated mice (n=4 per group). Histological scoring was performed in a blinded manner. Statistical differences were determined by one-way ANOVA with Tukey's multiple comparisons test. **p*<0.05. ****p*<0.001. Error bars represent SEM. Each symbol represents an individual mouse.

**
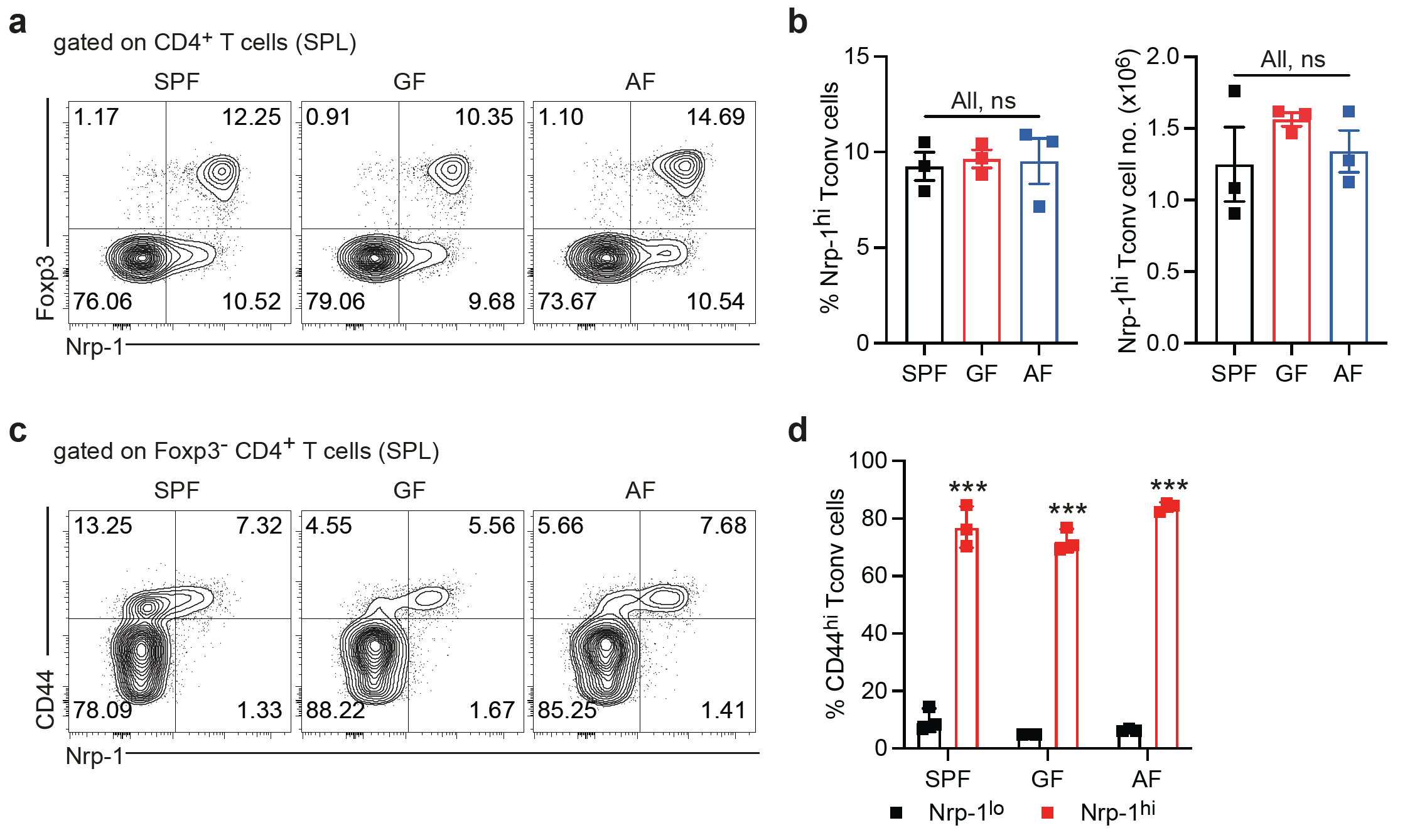
**

**Supplementary Fig. 6. Nrp-1^hi^ CD4^+^ conventional T cells are induced independently of foreign antigens.** Single cell suspensions were prepared from the spleen of 8-week-old specific pathogen-free (SPF), germ-free (GF), and antigen-free (AF) mice (n=3 per group). (a) Representative contour plot of Foxp3 and Nrp-1 gated on CD4^+^ T cells. (b) Frequency of Nrp-1^hi^ Tconv cells in CD4^+^ T cells (left) and total number of Nrp-1^hi^ CD4^+^ Tconv cells (right) in spleen of the indicated mice. (c) Representative contour plot of CD44 and Nrp-1 gated on CD4^+^ Tconv cells in the indicated mice. (d) The frequency of CD44^hi^ cells in Nrp-1^lo^ CD4^+^ Tconv and Nrp-1^hi^ CD4^+^ Tconv cells in the spleen of indicated mice. Two independent experiments showed similar results. Statistical differences were determined by one-way ANOVA with Tukey's multiple comparisons test (b) or two-way ANOVA with Bonferroni multiple comparisons test (c). ****p*<0.001, ns: not significant. Error bars represent SEM. Each symbol represents an individual mouse.

**
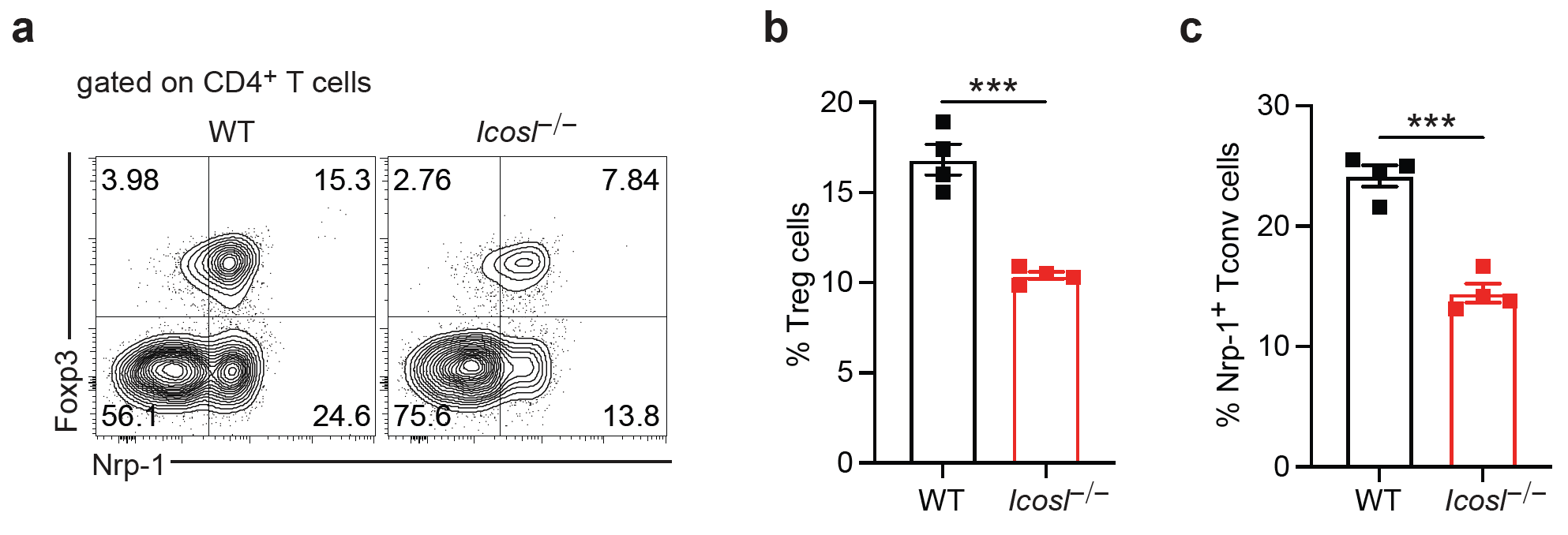
**

**Supplementary Fig. 7. Treg cells and Nrp-1^hi^ CD4^+^ conventional T cells are diminished in ICOSL-deficient mice.** Splenocytes of 16-week-old female B6 and ICOSL-deficient (*Icosl*^−/−^) mice were analysed (n=4 per group). (a) Representative contour plots of Foxp3 and Nrp-1 gated on CD4^+^ T cells. (b-c) Frequency of Treg cells (b) and Nrp-1^hi^ CD4^+^ Tconv cells (c) gated on CD4^+^ T cells in indicated mice. Statistical differences were determined by unpaired two-tailed Student’s *t* test. ****p*<0.001. Error bars represent SEM. Each symbol represents an individual mouse.

**
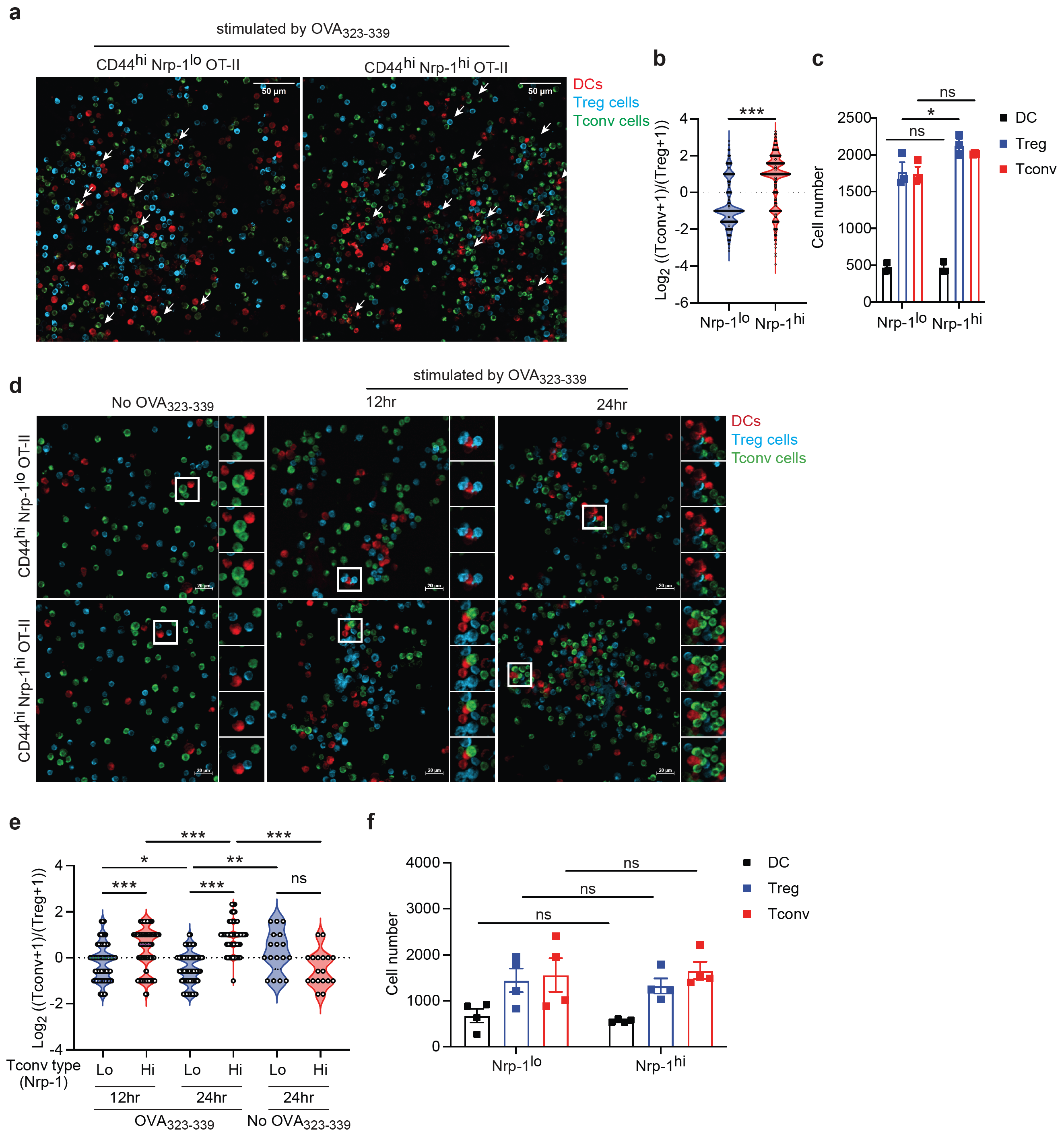
**

**Supplementary Fig. 8. Nrp-1^hi^ CD4^+^ conventional T cells exhibit enhanced DC engagement despite sharing antigen specificity with Treg cells.** (a-c) Lin^−^ CD11c^+^ dendritic cells (DCs) were co-cultured with Treg cells and either Nrp-1^lo^ or Nrp-1^hi^ CD44^hi^ CD4^+^ Tconv cells isolated from OT-II Transgenic mice. DCs, Treg cells, either Nrp-1^lo^ or Nrp-1^hi^ CD44^hi^ OT-II Tconv cells were labeled with lipophilic, fluorescent tracers, DiO, DiI and DiD, respectively. Cells were cultured for 24 hours. For antigenic stimulation, cells were treated with OVA_323-339_ peptides. Fluorescent signals were pseudocolored for visualization as follows: DCs, red; Treg cells, light blue; either Nrp-1^lo^ or Nrp-1^hi^ CD44^hi^ OT-II Tconv cells, green. (a) Representative immunofluorescence images showing interactions between DCs and either Nrp-1^lo^ (left) and Nrp-1^hi^ (right) OT-II Tconv cells in the presence of OT-II Treg cells. Arrows indicate DCs that preferentially interact with either Nrp-1^lo^ or Nrp-1^hi^ OT-II Tconv cells. Scale bar, 50 µm. (b) Violin plot showing the ratio of Nrp-1^lo^ and Nrp-1^hi^ CD44^hi^ OT-II Tconv cells to Treg cells among cells in close proximity to DCs. (c) Numbers of each T cell subset at the imaging time point. (d-f) DC-T interactions between DCs and either Nrp-1^lo^ or Nrp-1^hi^ OT-II Tconv cells in the presence of OT-II Treg cells were examined in the absence or presence of OVA_323-339_ peptide at 12 hr or 24 hr co-culture. (d) Representative immunofluorescence images showing interactions between DCs and either Nrp-1^lo^ (upper panel) and Nrp-1^hi^ (lower panel) OT-II Tconv cells versus OT-II Treg cells in the absence or presence of antigenic stimulation. The large image panels show representative fields of view. The adjacent smaller panels display corresponding z-stack images of the enclosed region in the large images, acquired at 1 µm intervals across the central region of the cells to confirm direct cell–cell contact in three dimensions. Scale bar, 20 µm. (e) Violin plot showing the ratio of Nrp-1^lo^ and Nrp-1^hi^ CD44^hi^ OT-II Tconv cells (lower panel) to OT-II Treg cells among cells in close proximity to DCs. (f) Numbers of each cell subset at 24 hours of co-culture. Statistical differences were determined by unpaired two-tailed Student’s *t* test (b), two-way ANOVA with Bonferroni multiple comparisons test (c, f) or one-way ANOVA with Tukey's multiple comparisons test (e). **p*<0.05. ***p*<0.01. ****p*<0.001. ns: not significant. Error bars represent SEM. Each symbol represents a biological replicate.

**
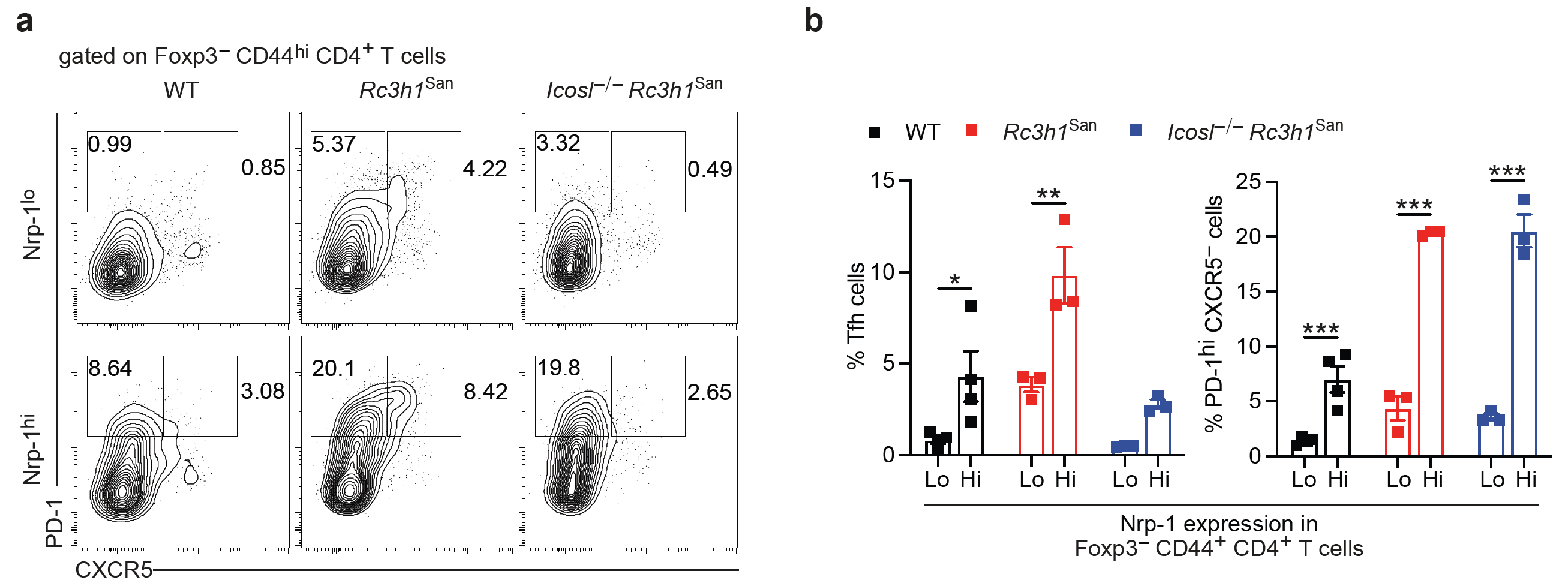
**

**Supplementary Fig. 9. PD-1^hi^ CXCR5+ and PD-1^hi^ CXCR5^−^ CD4^+^ conventional T cells are preferentially enriched in Nrp-1^hi^ CD4^+^ conventional T cells.** Splenocytes were prepared from 16-week-old WT, *Sanroque* (*Rc3h1*^San^) and ICOSL-deficient *Sanroque* (*Icosl*^−/−^ *Rc3h1*^San^) mice (n=4 per WT, n=3 per *Sanroque* and ICOSL-deficient *Sanroque* mice). (a) Representative contour plots showing PD-1 and CXCR5 expression on Nrp-1^lo^ (upper panel) or Nrp-1^hi^ (lower panel) CD44^hi^ CD4^+^ Tconv cells. (b) Frequency of PD-1^hi^ CXCR5^+^ Tfh cells (left) and PD-1^hi^ CXCR5^−^ Tph cells (right) in Nrp-1^lo^ and Nrp-1^hi^ CD44^hi^ CD4^+^ Tconv cells. Statistical differences were determined by two-way ANOVA with Bonferroni multiple comparisons test. **p*<0.05. ***p*<0.01. ****p*<0.001. Error bars represent SEM. Each symbol represents an individual mouse.

**
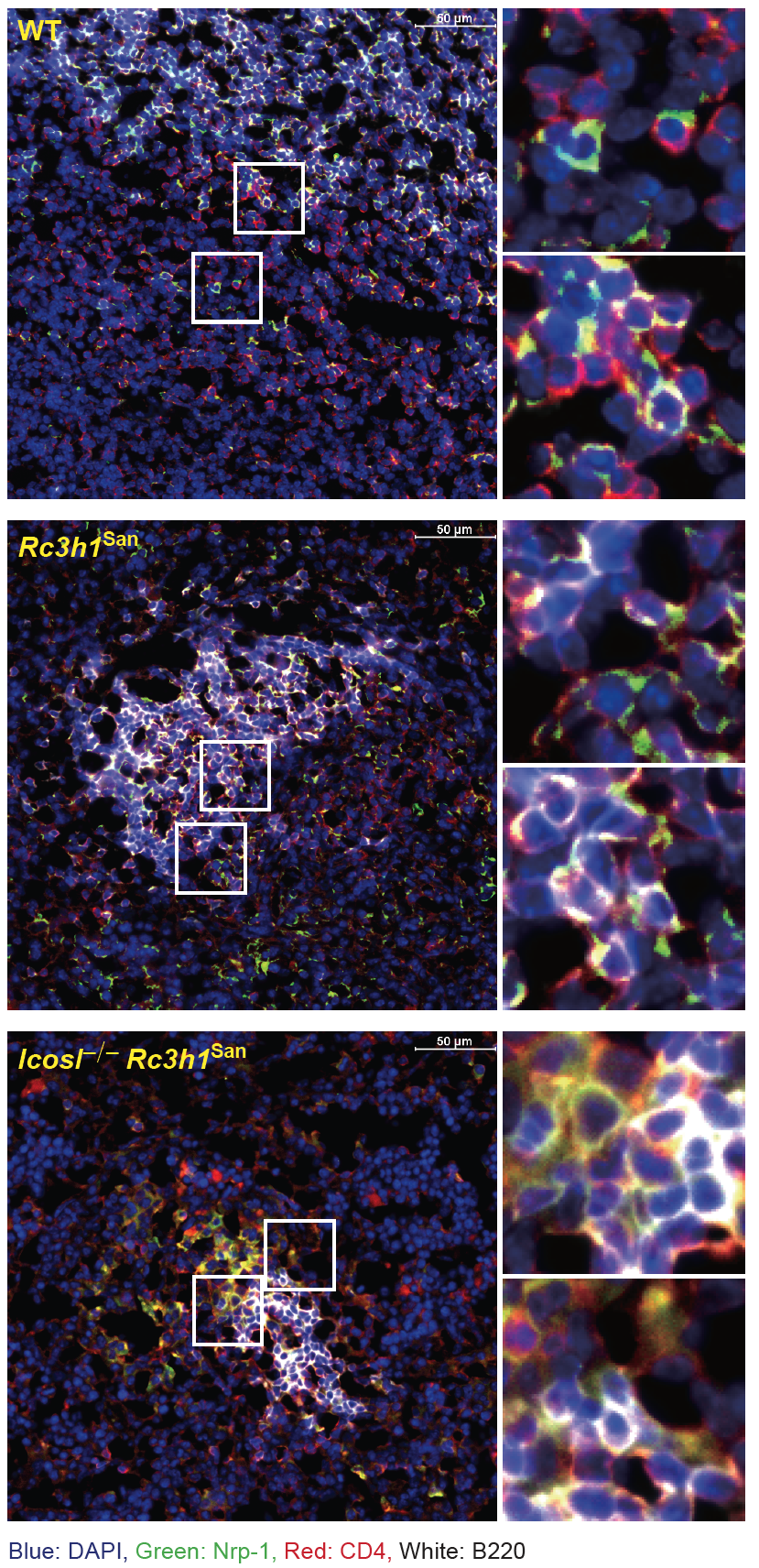
**

**Supplementary Fig. 10. Nrp-1^hi^ CD4^+^ conventional T cells are co-localized with B cells in ICOSL-deficient *Sanroque* mice.** Representative immunofluorescence image of spleen sections from the indicated mice showing Nrp-1^hi^ CD4^+^ T cells and B cells (left). The boxed regions were shown as magnified views (right). The scale bar indicates 50μm. DAPI is shown in blue, B220 in white, Nrp-1 in green and CD4 in red color.

**
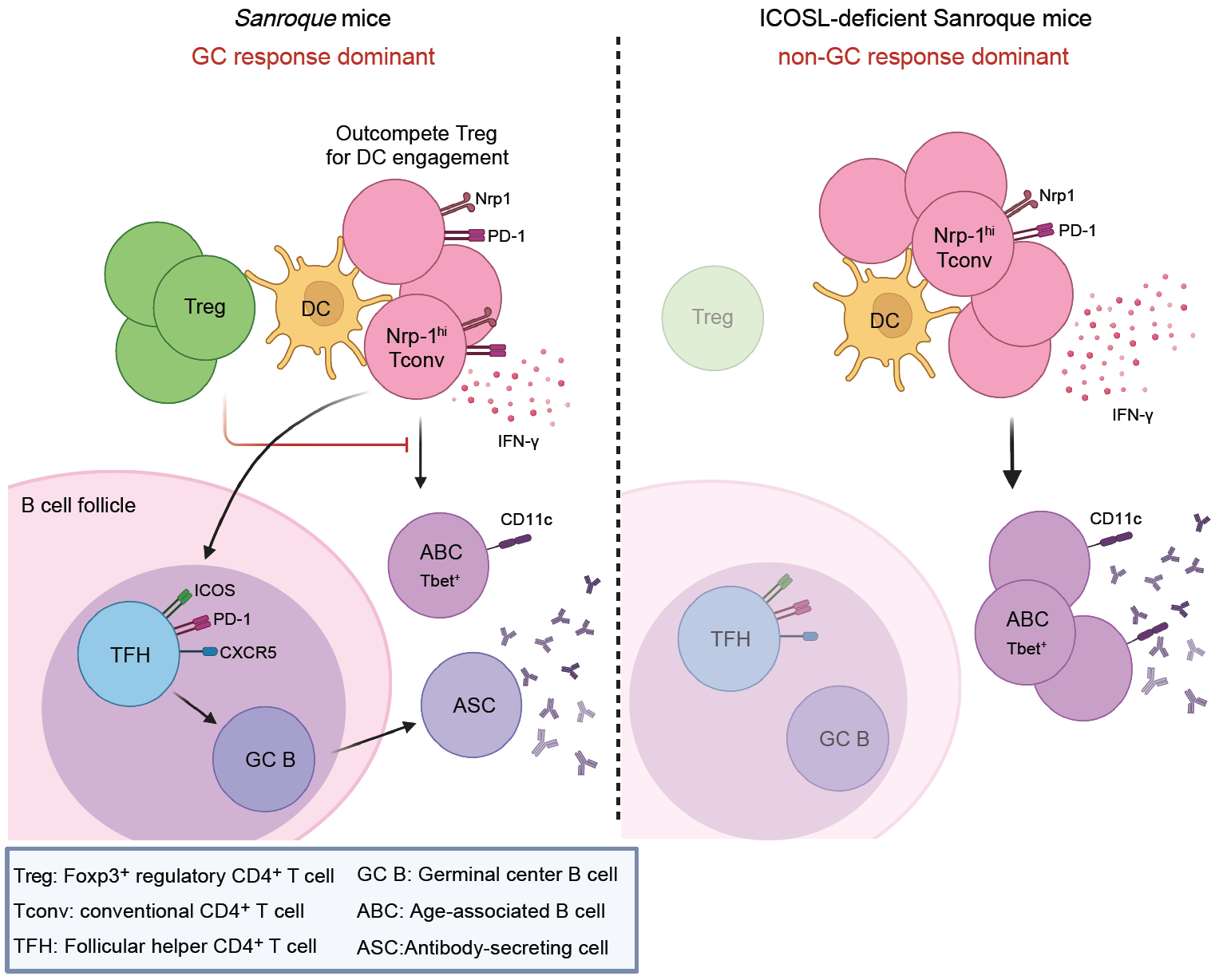
**

**Supplementary Fig. 11. Schematic diagram showing the pivotal role of ICOS signalling in regulating Treg cells and orchestrating GC-dependent and -independent B cell responses.** Created in BioRender. Kim, K. (https://BioRender.com/bd3r36q).
